# Quantifying cooperative FA^+^/MA^+^ ion migration in mixed perovskites via nano-infrared imaging

**DOI:** 10.1093/nsr/nwag071

**Published:** 2026-02-02

**Authors:** Jing Liang, Mu-Hao Lan, Shu Ding, Xing-Hua Xia, Jian Li

**Affiliations:** State Key Laboratory of Analytical Chemistry for Life Sciences, School of Chemistry and Chemical Engineering, Nanjing University, Nanjing 210023, China; State Key Laboratory of Analytical Chemistry for Life Sciences, School of Chemistry and Chemical Engineering, Nanjing University, Nanjing 210023, China; State Key Laboratory of Analytical Chemistry for Life Sciences, School of Chemistry and Chemical Engineering, Nanjing University, Nanjing 210023, China; State Key Laboratory of Analytical Chemistry for Life Sciences, School of Chemistry and Chemical Engineering, Nanjing University, Nanjing 210023, China; State Key Laboratory of Analytical Chemistry for Life Sciences, School of Chemistry and Chemical Engineering, Nanjing University, Nanjing 210023, China

**Keywords:** infrared photo-induced force microscopy, mixed A-cation perovskites, migration dynamics quantification, cooperative ion migration

## Abstract

Ion migration plays a critical role in the operational stability and efficiency of hybrid perovskite solar cells, yet direct and species-specific quantification of mobile cations remains challenging. Here, we employ infrared photo-induced force microscopy (IR-PiFM) to investigate the migration dynamics of MA^+^ and FA^+^ in MAPbI_3_, FAPbI_3_ and FA_0.5_MA_0.5_PbI_3_. These techniques allow nanoscale mapping and real-time tracking of individual cations with high chemical specificity and 250 μs temporal resolution. Our results reveal composition- and location-dependent ion transport behavior; while MA^+^ exhibits higher mobility in pure MAPbI_3_, its diffusion is significantly suppressed in the mixed-cation system. Surprisingly, FA^+^ and MA^+^ in FA_0.5_MA_0.5_PbI_3_ exhibit similar migration rates, suggesting a cooperative migration mechanism. Density functional theory calculations support this interpretation, showing that A-site mixing alters lattice symmetry and raises migration barriers through steric and electrostatic interactions. Bias-dependent nano-infrared imaging and open-circuit voltage measurements further show that mixed-cation perovskites confine cation motion and suppress bulk migration, leading to stabilized internal fields and improved voltage performance. These results provide direct evidence for cooperative cation migration dynamics in mixed A-cation perovskites and demonstrate that controlled cation mobility, rather than complete immobilization, may be key to achieving both efficiency and operational durability in perovskite optoelectronics.

## Teaser

Nano-infrared imaging reveals how two common organic ions move cooperatively inside mixed perovskites, offering a mechanistic basis for engineering perovskite devices with improved operational stability.

## INTRODUCTION

Metal halide perovskite solar cells are among the most promising candidates for next-generation photovoltaic technologies, offering certified power conversion efficiencies exceeding 27.3% alongside low-cost, solution-based fabrication routes [1–3]. These advances are largely driven by precise compositional and structural engineering of the perovskite lattice (ABX_3_), particularly at the A-site [4,5]. In this framework, divalent B-site cations (e.g. Pb^2+^) and halide X-site anions (I^−^, Br^−^, Cl^−^) form a BX_6_ octahedral network, while the A-site is populated by organic (methylammonium, MA^+^; formamidine, FA^+^) or inorganic (Cs^+^) cations that stabilize the structure via steric and hydrogen-bonding effects [6,7]. Mixed A-site cation formulations (MA^+^/FA^+^/Cs^+^) have been shown to enhance phase stability, suppress halide migration and improve both device efficiency and operational stability [8–10], yet the fundamental mechanisms underlying these improvements, especially the role of dynamic A-site cation migration, remain poorly understood.

Recent studies suggest that MA^+^ and FA^+^ exhibit markedly different ion transport behaviors in mixed-cation systems. MA^+^ (ionic radius: 217 pm), due to its smaller size and weaker hydrogen bonding (rotational barrier: 48.4 meV), can overcome a migration barrier of >0.5 eV under light or electrical bias [11–13], with a diffusion coefficient reaching up to 10^−8^ cm^2^/s, surpassing even that of halide ions [14]. This high mobility leads to MA^+^ accumulation at grain boundaries, triggering interfacial outgassing (e.g. MA^+^ → CH_3_NH_2_↑ + H_2_↑) and electrode corrosion [15]. In contrast, FA^+^ (ionic radius: 253 pm) exhibits stronger hydrogen bonding (rotational barrier: 95.0 meV) and steric hindrance, resulting in higher activation energies (0.55–0.99 eV) and largely irreversible migration behavior [16,17]. Notably, cooperative migration in mixed A-cation systems may establish a dynamic equilibrium: moderate Cs^+^ doping (5%–15%) can compress the lattice and reinforce FA^+^–X^−^ hydrogen bonding, thereby suppressing ion migration [18], whereas excessive Cs^+^ (>15%) induces Cs-rich secondary phases and lattice distortion [19]. These intricate effects highlight the limitations of conventional static compositional optimization strategies, underscoring the need to uncover atomistic migration pathways and coupling mechanisms of MA^+^ and FA^+^.

Conventional techniques for probing cation migration face three major limitations: (i) spatial resolution: macroscopic methods such as electrochemical impedance spectroscopy (EIS) and thermally stimulated current (TSC) cannot distinguish individual MA^+^ and FA^+^ contributions, while micro-analytical tools like secondary ion mass spectrometry (SIMS) suffer from sputtering damage and isobaric interference [20–23]; (ii) temporal resolution: *ex situ* techniques such as X-ray photoelectron spectroscopy (XPS) and X-ray diffraction (XRD) capture only the end states of ion migration, missing dynamic processes and the reversible migration of up to 30% of A-site cations [24–26]; (iii) multi-field coupling: under realistic operating conditions involving simultaneous light, electric field and thermal stimuli, ion migration couples intricately with lattice strain and defect dynamics, which are difficult to track in real time using conventional tools. Overcoming these limitations requires *in situ*, nanoscale techniques capable of resolving spatiotemporal cation migration under operational conditions [27–29].

**Figure 1.**
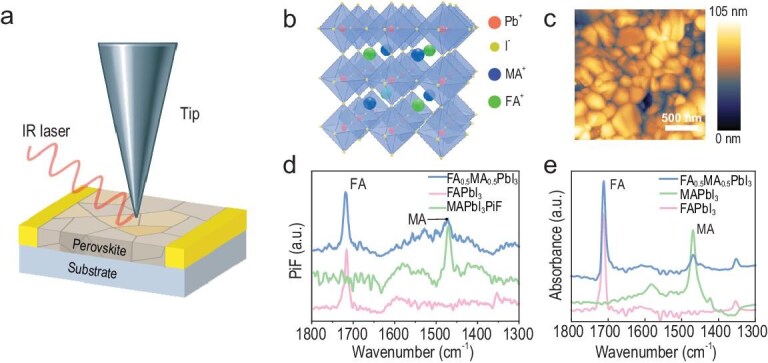
IR-PiFM. (a) Schematic of the IR-PiFM setup for perovskite measurements. (b) Illustration of the crystal structure of mixed-cation perovskites. (c) Topography of an FA_0.5_MA_0.5_PbI_3_ film measured by IR-PiFM, showing distinct grain and GB features. (d) IR-PiFM spectra measured on grain and (e) FTIR spectra of different mixed A-cation perovskite films.

In this study, we present a nanoscale infrared (nano-IR) strategy that enables *in situ*, spatially resolved and quantitatively traceable monitoring of A-site cation migration in mixed perovskite systems (Fig. 1). Nano-IR measurements reveal that the coexistence of MA^+^ and FA^+^ suppresses the diffusivity of both species relative to their single-cation counterparts, indicating reduced ionic mobility in the mixed phase. Complementary density functional theory (DFT) calculations and steady-state nano-imaging show that FA^+^ alters the migration pathway of MA^+^ through electrostatic shielding and steric hindrance, while acting as a kinetic bottleneck with increased activation energy. Furthermore, analysis of the correlation between cation migration and open-circuit voltage (*V*_oc_) establishes that compositional tuning of the A-site cations can effectively mitigate performance degradation. These findings offer a mechanistic framework for designing intrinsically stable, self-regulating mixed-cation perovskites. This nano-IR approach uniquely enables site-specific and species-specific quantification of cation dynamics in complex, mixed-A cation systems—a capability beyond the reach of conventional macroscopic or diffraction-limited techniques (for detailed comparison, see Table S1).

## RESULTS

To systematically investigate how A-site composition influences organic cation migration, we synthesized FAPbI_3_, MAPbI_3_ and FA_0.5_MA_0.5_PbI_3_ thin films via an antisolvent-assisted method. Scanning electron microscopy (SEM; Fig. S1) confirmed comparable grain sizes of 300–500 nm across all samples, minimizing the influence of grain boundary (GB) density on ion transport. FAPbI_3_ grains exhibited pronounced twin domains—planar defects more commonly observed in FA-rich compositions [30,31]. These features have previously been linked to anisotropic strain fields that may modulate local cation migration pathways. Steady-state photoluminescence (PL) spectra showed a slight redshift in peak emission from 797 nm (1.556 eV, MAPbI_3_) to 823 nm (1.506 eV, FAPbI_3_) with increasing FA^+^ content (Fig. S2), indicating minimal differences in bandgap (<0.05 eV) among the three compositions. This confirms that observed variations in ionic behavior primarily originate from A-site chemistry rather than electronic structure differences. To probe lateral ion transport under operational conditions, we fabricated planar devices with interdigitated gold electrodes spaced 50 μm apart (Fig. S3). This lateral geometry enables direct observation of field-driven ion migration along the film plane. All devices displayed enhanced photocurrent under illumination compared to dark current (Fig. S4), demonstrating robust photoconductive behavior and confirming the platform’s suitability for real-time cation migration analysis.

Temperature-dependent conductivity measurements [32], analyzed using the Arrhenius relation (Fig. S5), revealed an apparent activation energy of 0.499 ± 0.019 eV for FA_0.5_MA_0.5_PbI_3_, higher than those for MAPbI_3_ (0.389 ± 0.053 eV) and FAPbI_3_ (0.357 ± 0.017 eV). This trend is counterintuitive: the larger molecular size and stronger hydrogen bonding of FA^+^ (∼253 pm) should, in principle, confer a higher migration barrier than MA^+^ (∼217 pm). Paradoxically, ion transport appears more facile in FAPbI_3_ than in MAPbI_3_. This discrepancy likely stems from the inherent limitations of macroscopic conductivity measurements, which predominantly reflect the motion of majority carriers, typically halide ions, while underrepresenting minority species such as organic cations. Transient capacitance analysis (TCA), which can selectively probe ionic contributions, consistently reports higher activation energies for cation migration than conductivity-derived values [33], supporting the notion that standard conductivity underestimates organic ion mobility barriers. However, despite its improved selectivity, TCA remains a bulk-scale technique with spatial resolution limited to the micron scale. This constraint is particularly problematic for mixed A-cation perovskites, where nanoscale heterogeneity in phase distribution and defect structure can profoundly influence ion dynamics [34]. Moreover, all conventional electrical techniques inherently convolve the motion of multiple ionic species, making it impossible to distinguish contributions of specific cations.

To overcome these intrinsic limitations, we next employed infrared photo-induced force microscopy (IR-PiFM) [35–38]—a technique that detects chemical-bond absorption via photothermo-induced van der Waals forces (Fig. 1a). IR-PiFM provides two complementary detection modes with distinct spatial sensitivities: the heterodyne mode, which is surface-selective; and the homodyne mode, which integrates signals from both surface and subsurface layers [38]. Given that ion migration in perovskite films can vary markedly between surface and bulk regions, appropriate mode selection is critical. For films thicker than 500 nm with grain sizes of 300–500 nm, the homodyne mode offers sufficient spatial resolution to resolve grain interiors and boundaries. Despite some potential loss in lateral resolution due to thermal diffusion, this mode remains suitable for visualizing bulk ion transport.

Before conducting migration measurements, we performed comprehensive pre-characterization of the perovskite films to validate the chemical specificity and spatial resolution of our IR-PiFM platform. As shown in Fig. 1c, the atomic force microscopy (AFM) topography reveals uniform grains with typical sizes of 300–500 nm, showing distinct grain and GB features similar to the SEM observations. To further validate the chemical specificity of IR-PiFM, we compared the nano-IR spectra of MAPbI_3_, FAPbI_3_ and FA_0.5_MA_0.5_PbI_3_ (Fig. 1d) with their corresponding Fourier-transform infrared (FTIR) spectra (Fig. 1e). MAPbI_3_ displayed characteristic peaks at 1468 cm^−^^1^ (NH_3_^+^ symmetric bending) and 911 cm^−^^1^ (CH_3_/NH_3_ rocking), while FAPbI_3_ exhibited a prominent peak at 1714 cm^−^^1^ associated with the C–N antisymmetric stretch of FA^+^ [39]. In the mixed A-cation perovskite, all the three absorption bands appear, showing similarity between grain boundaries and grains (Fig. S6). The strong spectral agreement between the IR-PiFM and FTIR results confirms its high chemical identification capability in distinguishing MA^+^ and FA^+^. Notably, in the mixed system, the FA^+^ signal was approximately 2.3 times stronger than the MA^+^ signal, consistent with their known differences in infrared absorption cross-section. This further validates IR-PiFM as a reliable tool for tracking the spatial distribution of individual A-site cations.

**Figure 2.**
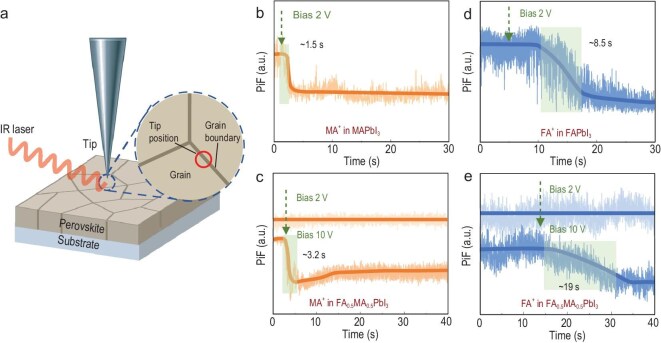
(a) Schematic of ‘single-point, single-wavelength’ IR-PiF detection. (b–e) Bias-induced temporal resolved IR-PiF signal curves of cations in GB: MA^+^ in (b) MAPbI_3_ and (c) FA_0.5_MA_0.5_PbI_3_ films; FA^+^ in (d) FAPbI_3_ and (e) FA_0.5_MA_0.5_PbI_3_ films. The laser wavenumber was set as 1468 cm^−^^1^ for MA^+^ migration, and 1714 cm^−^^1^ for FA^+^ migration. The solid lines in (b–e) serve solely as visual guides to aid in following the signal evolution trend, without implying any specific quantitative relationship.

To overcome the temporal limitations of imaging-based techniques, we developed a ‘single-point, single-wavelength, fixed-position tracking’ strategy using infrared photo-induced force (IR-PiF) detection (Fig. 2a) [40]. Based on preliminary spectral analysis (Fig. 1d), the excitation wavelength of the quantum cascade laser (QCL) was locked to the MA^+^-specific 1468 cm^−^^1^ mode or the FA^+^-specific 1714 cm^−^^1^ mode, thereby avoiding time-consuming full-spectrum scanning.

After scanning the surface morphology via AFM, the probe was positioned with nanometer precision (±5 nm) at designated sites, either the grain center or GB. With electrode spacing of 50 μm, the applied biases (e.g. 2 V, 10 V) correspond to average electric fields of 0.04 and 0.2 V/μm, respectively. These field strengths are comparable to, or even milder than, the built-in fields present in operational vertical perovskite solar cells (typically on the order of ∼1 V/μm). Thus, our experiments probe cation migration under relevant driving forces. The key findings pertain to the relative migration rates, preferential pathways and coupling mechanisms between different A-site cations, which are intrinsic material properties and are elucidated under these practically relevant conditions. IR-PiF signals were continuously recorded under an applied bias using a customized high-speed acquisition module with a signal acquisition frequency of ∼4000 Hz (time resolution: ∼250 μs; Fig. S7). This represents a six-order-of-magnitude improvement over conventional nano-IR imaging (∼6 min per frame). Baseline drift remained below 3% over 40 min of zero-bias acquisition (Figs S8 and S9), firmly establishing the reliability of *in situ* measurements. In addition, potential interference from laser power on the signal was eliminated by PiFM measurements conducted under different laser powers (Figs S10 and S11).

Time-resolved IR-PiF measurements revealed distinct cation migration kinetics across different compositions. At the GB of MAPbI_3_, a 2 V bias induced a 50% reduction in the MA^+^ signal within 1.5 s (Fig. 2b), corresponding to a drift velocity of approximately 33.33 μm/s, which becomes slower for FA^+^ in FAPbI_3_ at 5.88 μm/s (Fig. 2d). In FA_0.5_MA_0.5_PbI_3_, a higher bias of 10 V was required to achieve a 40% reduction in MA^+^ signal over 3.2 s (Fig. 2c), while FA^+^ migration proceeded even more slowly, with a 40% decrease occurring over 19 s (Fig. 2e). These results highlight the suppressed mobility of both cation species in the mixed system, with FA^+^ being particularly sluggish.

Given a defined bias, the separation distance between the two Au electrodes, and the observed time required for ion migration to reach equilibrium, the drift velocities of specific cations were calculated. Species-specific mobilities and diffusion coefficients were then estimated using the Einstein relationship (see calculation details in Section S13 and Table S2). Averaging the results from different devices for each case (Section S15), the MA^+^ diffusion coefficient in MAPbI_3_ was 2.05 × 10^−7^ cm^2^/s, decreasing to 1.36 × 10^−8^ cm^2^/s in the mixed composition. The FA^+^ diffusion coefficient was even lower in FA_0.5_MA_0.5_PbI_3_, at 3.25 × 10^−9^ cm^2^/s, consistent with its higher migration barrier inferred from macroscopic conductivity measurements (Fig. S5).

Importantly, time-resolved IR-PiF spectra acquired within grain interiors showed negligible signal decay under both 2 V and 10 V bias conditions (Figs S15–S17), indicating that operational cation migration is predominantly confined to grain boundaries [41,42]. These findings underscore the power of IR-PiF as a chemically selective, high-resolution platform for quantifying spatially dependent cation dynamics in heterogeneous perovskite systems.

Interestingly, while the absolute diffusion coefficients of FA^+^ and MA^+^ in FA_0.5_MA_0.5_PbI_3_ are shown to be different, they exhibit synchronized migration, suggesting comparable activation energies. This behavior contradicts conventional expectations based on lattice size; XRD results show that FA incorporation enlarges the A-site cavity, which should, in principle, facilitate MA^+^ migration while hindering FA^+^ (Fig. S18). Experimentally, however, although FA^+^ mobility decreases as expected, MA^+^ migration also slows significantly—its diffusion coefficient drops by an order of magnitude. This counterintuitive result indicates that MA^+^ transport is not governed solely by lattice geometry, but is strongly modulated by the presence and mobility of FA^+^.

We therefore propose a cooperative migration mechanism, in which MA^+^ and FA^+^ move through shared ionic pathways, with the motion of one species facilitating the other. When FA^+^ becomes immobilized, it imposes steric and electrostatic hindrance that obstructs MA^+^ transport.

**Figure 3.**
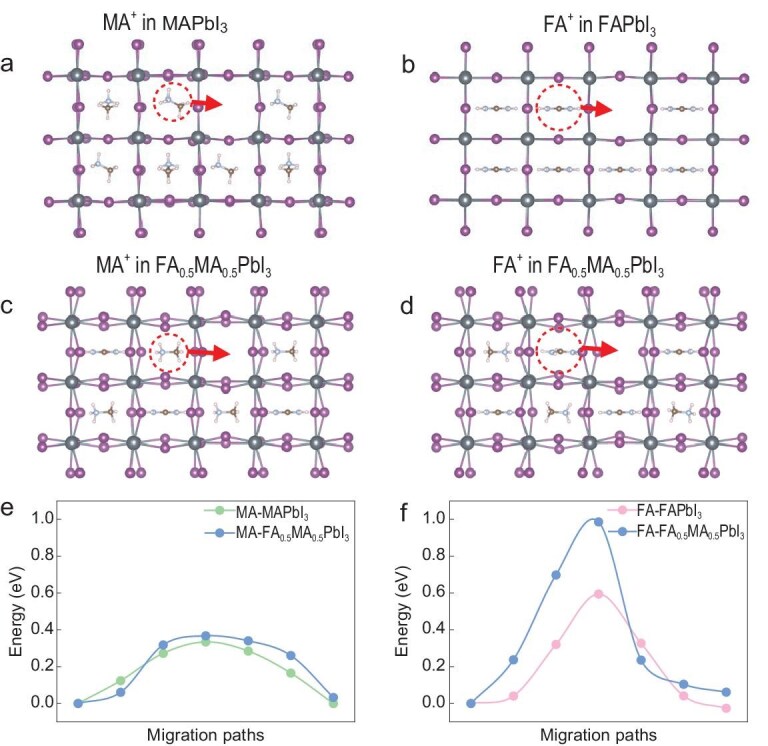
Calculated relative activation energies for the A-cation migration. (a–d) Crystal structures of MAPbI_3_ (a), FAPbI_3_ (b) and FA_0.5_MA_0.5_PbI_3_ (c and d). The red arrows represent the different cation migration pathways. (e) Calculated relative activation energies for the MA^+^ migration in MAPbI_3_ and FA_0.5_MA_0.5_PbI_3_, respectively. (f) Calculated relative activation energies for the FA^+^ migration in FAPbI_3_ and FA_0.5_MA_0.5_PbI_3_, respectively.

To test this hypothesis, we performed DFT calculations using 4 × 2 × 2 supercell models of FAPbI_3_, MAPbI_3_ and FA_0.5_MA_0.5_PbI_3_. Migration pathways were constructed using the climbing-image nudged elastic band (Cl-NEB) method (Fig. 3a–d, Figs S19–S22) [32,43,44]. In MAPbI_3_, MA^+^ migrates through a tilted Pb–I plane with a relatively low activation energy (*E*_a_ = 0.334 eV) (Fig. 3e). In the mixed system, however, FA^+^ suppresses octahedral tilting, narrowing the migration channel cross-section by ∼34% and raising the *E*_a_ of MA^+^ to 0.367 eV (Fig. 3e). For FA^+^, the *E*_a_ increases more dramatically, from 0.595 eV in FAPbI_3_ to 0.987 eV in FA_0.5_MA_0.5_PbI_3_ (Fig. 3f), due to stronger early-stage interactions with neighboring I^−^ ions, leading to lattice distortion and higher energy penalties. These calculations support the notion that cation transport is dynamically coupled rather than independent.

We further validated the cooperative migration model through bias-dependent IR-PiFM imaging. By tuning the QCL to 1714 and 1468 cm^−^^1^, we selectively tracked FA^+^ and MA^+^ distributions, respectively. All imaging was performed in homodyne mode rather than heterodyne mode. While heterodyne mode offers superior surface sensitivity, the homodyne mode is uniquely suited for quantifying bulk cation migration within grains and across grain boundaries (Section S1). Each map was acquired after applying an external bias for 3 min (Fig. S23), which is sufficient to establish steady-state ion profiles. Control experiments under zero bias for 40 min showed no cation redistribution, ruling out imaging artifacts and confirming structural stability (Figs S8 and S9).

**Figure 4.**
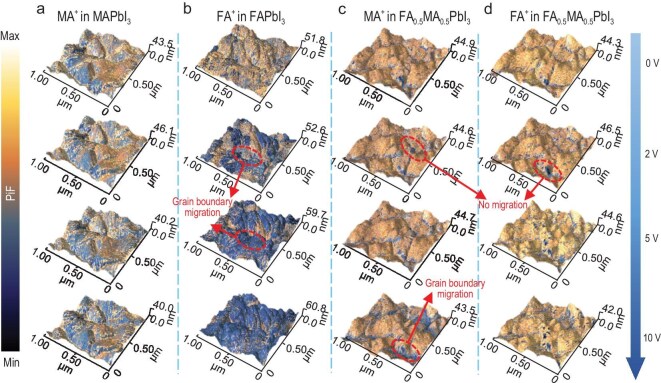
Nano-IR investigation of cation migration. Merged AFM and bias-dependent homodyne mode IR-PiFM images at 1468 cm^−^^1^ obtained on (a) MAPbI_3_ and (c) FA_0.5_MA_0.5_PbI_3_ films, and at 1714 cm^−^^1^ on (b) FAPbI_3_ and (d) FA_0.5_MA_0.5_PbI_3_ films, respectively. For FA_0.5_MA_0.5_PbI_3_ film, the IR-PiFM images were recorded on the same region by switching the IR wavenumber.

At low bias (2 V), IR-PiFM maps revealed composition- and geometry-dependent migration behaviors (Fig. 4a and b). In MAPbI_3_ and FAPbI_3_, GB regions exhibited significant signal depletion, while grain interiors remained largely unchanged, indicating GB-dominated transport. In contrast, the mixed perovskite showed minimal signal change in both regions (Fig. 4c and d), and the spatial profiles of FA^+^ and MA^+^ were closely aligned. This synchronized migration front reinforces the hypothesis of cooperative, channel-sharing transport, which is further confirmed by the similar observation over different devices (Section S21).

To simulate operating conditions more realistically, we applied higher biases (5 and 10 V), comparable to internal electric fields in working devices (>1 V/μm) [45]. Under these conditions, both MAPbI_3_ and FAPbI_3_ exhibited strong cation depletion within grains (Fig. 4a and b), indicating a transition from GB-limited to bulk migration. However, in FA_0.5_MA_0.5_PbI_3_ (Fig. 4c and d), cation signals remained relatively uniform, with only moderate depletion at GBs. The uniform decrease of cation signal is further verified by analyzing the extracted line profile at the marked grain boundaries position (Figs S27 and S28). These results suggest that A-site mixing not only raises migration barriers, but also spatially confines ion transport, particularly within the grain interior. Additionally, the bias-dependent heterodyne mode IR-PiF maps of FA_0.5_MA_0.5_PbI_3_ also show that the surface migration of MA^+^ and FA^+^ also occurs at bias higher than 5 V (Fig. S29), indicating that the cation composition-engineering is also beneficial to suppressing the migration in surface channels.

To bridge nanoscale ion dynamics with device-level behavior, we analyzed how cation migration under an applied electric field affects the internal potential of perovskite devices. Although our experiments do not directly measure light-induced *V*_OC_ [46], we employed a bias-induced *V*_OC_ to reflect changes in built-in field strength caused by ion redistribution (Fig. S30). This approach enables us to assess how compositional variations modulate ionic screening effects under operating-like conditions.

**Figure 5.**
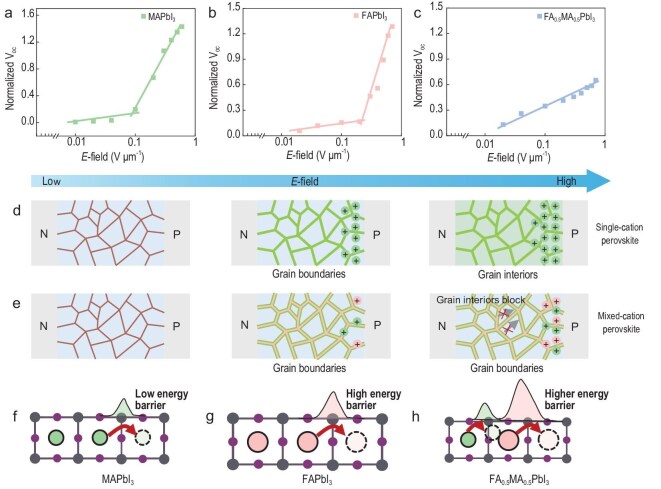
(a–c) *V*_OC_ values of the different devices fabricated with (a) MAPbI_3_, (b) FAPbI_3_ and (c) FA_0.5_MA_0.5_PbI_3_ perovskite films under different biases. Polling time for each measurement: 100 s. Schematic diagram of cation migration pathways in (d) single-cation perovskite and (e) mixed-cation perovskite. The green and pink dots respectively represent MA^+^ and FA^+^; brown line: GBs; green areas: migration pathways of single cation. Schematic diagram of lattice structure and cation migration of (f) MAPbI_3_, (g) FAPbI_3_ and (h) FA_0.5_MA_0.5_PbI_3_.

As demonstrated by IR-PiFM imaging and IR-PiF kinetics, MAPbI_3_ and FAPbI_3_ exhibit prominent cation migration along GBs at low fields and into the grain interior under higher bias (Fig. 5a–c), consistent with a transition from GB- to bulk-mediated transport. Correspondingly, their *V*_OC_-bias profiles show two-stage behavior: a gradual rise at low voltage followed by a sharp increase beyond a threshold, marking the onset of bulk migration and enhanced ionic screening. In contrast, the mixed perovskite FA_0.5_MA_0.5_PbI_3_, characterized by cooperative A-site cation migration and suppressed bulk transport, exhibits a smooth, monotonic *V*_OC_ increase across the full bias range, suggesting more uniform and restrained ion dynamics (Fig. 5c).

Taken together, our IR-PiFM imaging and *V*_OC_ measurement highlight that A-site cation mixing not only increases the activation barrier for migration, but also modulates the spatial migration pathway. In the mixed system, FA^+^ and MA^+^ migrate in a synchronized, cooperative manner through shared pathways. These cooperative interactions between MA^+^ and FA^+^ ions increase the migration activation energy and reduce their mobility, particularly within the grain interior (Fig. 5d and e). In terms of structure, mixed A-cation perovskite exhibits lattice contraction compared to FA^+^/MA^+^ single-cation perovskite (Fig. 5f–h and Section S18). In mixed A-cation perovskites, lattice contraction elevates the activation energy for FA^+^ migration and concurrently influences MA^+^ migration through FA^+^ coupling, which has been fully confirmed by our experiments and DFT calculation.

As a result, ionic screening of the electric field is mitigated, leading to improved field retention and more stable potential profiles under bias.

Interestingly, although bulk migration is largely suppressed in FA_0.5_MA_0.5_PbI_3_, the continuous *V*_OC_ rise under bias suggests that moderate GB-level migration still persists. This behavior reflects a well-regulated ion migration regime that allows partial field modulation without inducing excessive ionic redistribution. Such balanced ionic mobility may provide the optimal trade-off between operational field stability and device longevity.

## CONCLUSION

In this study, we conducted a multi-scale investigation of A-site cation migration in hybrid perovskites, integrating macroscopic activation energy analysis, nano-IR imaging and device-level measurements. By employing IR-PiFM and time-resolved IR-PiF spectroscopy, we achieved spatially and temporally resolved tracking of FA^+^ and MA^+^ cations under applied electric fields, enabling species-specific quantification of their migration behaviors within individual grains.

Our results reveal that MA^+^ and FA^+^ exhibit distinct mobilities and field-induced dynamics, even in a chemically mixed lattice. Notably, in FA_0.5_MA_0.5_PbI_3_, the two cations display nearly identical diffusion coefficients and synchronized spatial migration fronts, suggesting a cooperative migration mechanism. This hypothesis was further supported by DFT calculations, which showed that the presence of FA^+^ modifies the MA^+^ migration pathway by suppressing octahedral tilting and increasing the associated activation barrier.

Bias-dependent nano-IR imaging revealed that A-site cation mixing not only elevates thermodynamic migration barriers but also spatially confines ion transport, particularly suppressing bulk migration. This behavior was reflected in device-level bias-induced *V*_OC_ measurements, where mixed-cation perovskites displayed a smooth voltage evolution and improved *V*_OC_ stability, in contrast to the more abrupt transitions observed in MAPbI_3_ and FAPbI_3_.

Collectively, these findings demonstrate that ion migration in hybrid perovskites is composition-dependent, spatially heterogeneous and dynamically coupled. Our work highlights the power of nano-IR spectroscopy for probing ionic motion with chemical specificity and spatial precision, and underscores the potential of A-site engineering in modulating ion transport pathways to optimize perovskite solar cell performance and stability. While this study elucidates the cooperative migration between FA^+^ and MA^+^, the underlying mechanism—where A-site cation diversity introduces steric and electrostatic interactions that hinder ion diffusion—may be a general design principle. The present approach could be extended to more complex compositions, including those incorporating inorganic cations like Cs^+^. In such systems, the static lattice stabilization provided by Cs^+^ may synergize with the dynamic coupling between organic cations, offering a multi-faceted strategy for stability engineering.

## Supplementary Material

nwag071_Supplemental_File
