# Supplementary material for: Quantifying cooperative FA+/MA+ ion migration in mixed perovskites via nano-infrared imaging
Source: Natl Sci Rev. 2026 Feb 2;13(6):nwag071. doi: 10.1093/nsr/nwag071 (PMC13032879; doi:10.1093/nsr/nwag071)
Supplement: nwag071_Supplemental_File [file nwag071_supplemental_file.pdf]

Supporting Information

Quantifying Cooperative FA^+/MA^+ Ion Migration in Mixed Perovskites via Nano-Infrared Imaging

Jing Liang, Mu-Hao Lan, Shu Ding, Xing-Hua Xia*, Jian Li*

State Key Lab of Analytical Chemistry for Life Science, School of Chemistry and Chemical Engineering, Nanjing University, Nanjing 210023, China

Table of content

S1. Methods section

S2. Comparison of different nanoscale characterization techniques

S3. SEM images of different mixed A-cation perovskite films

S4. Steady-state photoluminescence (PL) spectra of different mixed A-cation perovskite films

S5. Schematic illustration of the lateral device for PiFM measurement

S6. I-V curves of the different mixed A-cation perovskite devices obtained in dark and under illumination

S7. Ionic migration activation energy of different mixed A-cation perovskite

S8 Comparison of IR-PiFM spectra between grain boundaries and grains.

S9. Signal acquisition frequency of temporal resolved IR-PiFM

S10. Long term stability of $FA_{0.5}MA_{0.5}PbI_3$ perovskite device under IR-PiFM imaging

S11. Long term stability of $FAPbI_3$ perovskite device under IR-PiFM imaging

S12. IR-PiF measurement in different laser power

S13. Calculated diffusion coefficient and comparison

S14. Time-dependent IR-PiF signal curve on grain boundaries of different perovskite devices

S15. Calculation and statistics of diffusion coefficient

S16. Time-dependent IR-PiF signal curve in on grains of differentperovskite devices

S17. XRD patterns of different A-cation perovskite

S18. DFT simulations for cation migration

S19. Time dependent IR-PiFM images of $FAPbI_3$ device under bias

S20. Bias cycling experiment of $FAPbI_3$ device

S21. IR-PiFM images of different perovskite devices before and after applying bias

S22. Line profile of AFM and external electric field dependent PiFM signals of cations in $FA_{0.5}MA_{0.5}PbI_3$ perovskite device

S23. Imaging of electric field induced cation migration on $FA_{0.5}MA_{0.5}PbI_3$ perovskite device via heterodyne mode

S24. Scheme of the set up for V_{OC} measurements

S1. Methods section

Perovskite synthesis and device fabrication

$FAPbI_3$ perovskite film

The glass substrates were ultrasonic precleaned with acetone, ethanol and deionized water respectively for 10 min. After washing, the substrates were dried with nitrogen, and then treated with ultraviolet ozone for 10 min. FAI and PbI_2 were dissolved in an anhydrous DMF with a stoichiometric ratio of 1:1, resulting in a perovskite precursor solution with a concentration of 1.3 M at room temperature.after adding HI (0.10 g, 57wt% water) to 1 mL of $FAPbI_3$, the 1.3M precursor solution was spin coated to the cleaned glass substrate at 6500 rpm for 30 s with an acceleration of 1300 rpm/s. A perovskite film with a thickness of ~500 nm was then deposited on the treated glass substrate. After starting spin coating for 3 s, 200 μL chlorobenzene was quickly dropped onto the surface of perovskite as an antisolvent. Then, the deposited precursor film was annealed at 150 °C for 20 min.[1]

$FA_{0.5}MA_{0.5}PbI_3$ perovskite film

MAI, FAI and PbI_2 were dissolved in anhydrous DMF with a stoichiometric ratio of 1:1:2, resulting in a perovskite precursor solution with a concentration of 1.3 M at room temperature. Then, the antisolvent method was used to spin the 1.3 M precursor solution onto the cleaned glass substrate at 6500 rpm for 30 s with an acceleration of 1300 rpm/s. A perovskite film with a thickness of ~500 nm was deposited. After starting spin coating for 3 s, 200 μL chlorobenzene was quickly dropped onto the surface of perovskite as an antisolvent. After spin coating, the deposited precursor film was annealed at 100 °C for 10-20 min. In our experiment, all cation compositions of perovskite films refer to chemical proportions in the precursor solution.[1]

$MAPbI_3$ perovskite film

To prepare a precursor solution with a concentration of 1.35 M, PbI_2 and CH_3NH_3I (molar ratio 1:1) were dissolved in DMF: DMSO (v:v = 9:1). Then this solution was spin-coated onto the cleaned glass substrate (first at 2000 rpm for 5 s, and then at

4000 rpm for 20 s). After starting the spin-coating for 7 s, 100 μL of methylbenzene as an anti-solvent was quickly dropped on the perovskite surface. Finally, the deposited precursor film was transferred to an electric hotplate at 100 ℃ for annealing over a period of 10 min.

XRD measurements

XRD measurements were carried out on a Shimadzu Lab X/XRD-6000 X-ray diffractometer equipped with a Cu-Kα radiation source (λ = 1.5418 Å) operating at 40 kV and 30 mA.

Photoluminescence (PL) measurements

Photoluminescence (PL) of different A-site cations perovskite films was measured by FLS980 (Edinburgh Instruments Ltd) with an excitation at 532 nm [2].

Devices characterization

Electrochemistry measurements were performed on lateral solar devices using a probe station (Lake Shore Cryotronics, Inc) chamber under a vacuum of 10^{-5} Pa in dark condition using a Keithley 2450 source meter.

Ion migration activation energy measurements

Ion migration activation energy was determined by measuring temperature-dependent conductivity change on the lateral devices under an applied average electrical field of 0.2 V/μm. The activation energy E_a was then extracted by fitting the raw data to the Arrhenius equation [3]:

$$\sigma(T) = Aexp(\frac{-E_a}{k_B T}) \quad \text{(S-1)}$$

where $\sigma(T)$ is temperature-dependent conductivity, A is pre-exponential constant, and k_B is Boltzmann's constant. The activation energy was calculated from the slope of the ln(σ)–1/($k_B T$) relation.

Open-circuit voltage measurements

The lateral devices were placed horizontally under different polarization electric fields and kept for 100 s. After removing the electric field, the potential difference between the two Au electrodes was measured using a MFLI (Zurich instruments).

FTIR and PiFM measurements

FTIR and PiFM measurements were conducted under dark condition and environmental atmosphere. FTIR spectra were collected on a Nicolet IS50 (Nicolet, USA), AFM and PiFM were obtained on a VistaScope system (Molecular Vista, USA). PiFM measurements were taken on a VistaScope microscope that is coupled to a quantum cascade laser (QCL) system (Block Engineering, USA) with 1 cm^{-1} wavenumber resolution (range: 770 to 1885 cm^{-1}). The *p*-polarized pulsed QCL was focused at the tip end of a platinum-iridium cantilever through an integrated off-axis parabolic mirror. The set point was set as 80% with an oscillation amplitude of ~1 nm. The cantilever was excited at its second resonance frequency around 1.43 MHz. The time per image was about 6 min at 256×256 resolution. The spectrum was normalized with the background laser power profile spectrum.

The PiFM system operated in both heterodyne and homodyne modes. The infrared photo-induced force in our perovskite samples is dominated by the photothermal expansion effect. The detected force F_{th} can be described as $F_{th}(z) \propto \Delta L/z^3$, where z is the tip-sample distance and ΔL is the sample thermal expansion.

The total thermal expansion ΔL_{tot} comprises two components: $\Delta L_{tot}(z)=\Delta L_t(z)+\Delta L_d$. The distance-independent term, ΔL_d , arises from the bulk photothermal expansion of the illuminated volume. In contrast, the distance-dependent term, $\Delta L_t(z)$, originates from the highly localized tip-enhanced optical field confined to the nanoscale region beneath the tip[4-5].

In PiFM, the heterodyne mode is configured to detect the gradient of the force, dF/dz. This gradient is predominantly sensitive to the z-dependent component, $\Delta L_t(z)$, making the heterodyne signal highly surface-sensitive. Conversely, the homodyne mode measures the total force F_{th}, which is largely governed by the bulk

contribution ΔLd . Consequently, the homodyne signal scales approximately linearly with the concentration of absorbing species (e.g., MA^+ or FA^+) within the probed volume, providing a quantitative measure of bulk cation distribution. This fundamental distinction guided our selection of the homodyne mode for monitoring bulk cation migration in grain interiors and boundaries.

While the heterodyne mode is ideal for probing surface chemistry due to its sensitivity to $\Delta L_t(z)$, our goal was to quantify cation migration within the bulk. Critically, as the perovskite surface is amorphous whereas the bulk is crystalline, homodyne detection is required to access lattice-specific information from the crystalline interior. The homodyne signal's linear scaling with the volume of excited material enables depth-integrated, quantitative analysis of cation concentration, making it uniquely suited for mapping bulk dynamics. This choice was essential to achieve spatially resolved, quantitative mapping of cation dynamics in the perovskite film bulk, which is the central methodological advance of this work. During bias-controlled experiments, the scanning direction was consistent with the electric field direction.

Computational details

All the calculations were performed in the framework of the density functional theory with the projector augmented plane-wave method, as implemented in the Vienna ab initio simulation package.[6] The generalized gradient approximation proposed by Perdew, Burke, and Ernzerhof was selected for the exchange-correlation potential.[7] The long range van der Waals interaction was described by the DFT-D3 approach.[8] The plane wave cut-off energy of 400 eV was adopted, the energy convergence accuracy was set to 1×10^{-6} eV/atom, and the force acting on each atom was not greater than 0.05 eV/Å. The Brillouin zone was integrated using a $2 \times 1 \times 1$ k-point grid.

The Climbing Image Nudged Elastic Band (Cl-NEB) method, which enables the determination of the minimum energy path between two energetically stable end points, was used to evaluate the value of ΔE_a, where $4 \times 2 \times 2$ cell of $MAPbI_3$ and $FAPbI_3$ was used. The final structures were constructed by removing MA or FA

molecule from the initial structures. We then carried out conventional geometry optimization with the force cutoff of 10^{-6} Ry/bohr. The Cl-NEB method was applied to a discretized path of with 7 images. We calculated the activation energies as the difference between the maximum and minimum energies of the calculated Cl-NEB images. Intermediate images were constructed by combination of rotation and translation of the migrating MA and FA molecules. The cell parameters were fixed at the optimized values for the perfect crystals. All the atoms of intermediate Cl-NEB images were relaxed, while atomic structures of the start and end points were always fixed in Cl-NEB calculations.

S2 Comparison of different nanoscale characterization techniques

Table S1. Comparison of nanoscale characterization techniquesfor probing ion

Technique	Imaging Mechanism	Spatial Resolution	Topography	Chemical Specificity	Signal Origin	Key Limitations for Cation Migration Studies
Photoluminescence (PL)	Optical emission	~500 nm	No	No (indirect defect sensing)	Bulk	No chemical ID; poor spatial resolution; indirect signal.
Kelvin Probe Force Microscopy (KPFM)	Surface potential	~50 nm	**Yes** (Height)	No (electrostatic potential only)	Surface	No chemical ID; sensitive to environment and surface states.
Conductive Atomic Force Microscopy (C-AFM)	Current	~50 nm	**Yes** (Height)	No (conductivity only)	Surface	No chemical ID; measures electronic current, not ion motion.
Secondary Ion Mass Spectrometry (SIMS)	Mass spectrometry	~100 nm	No	**Yes (elements/isotopes)**	Surface (destructive)	**Destructive**; poor spatial resolution; difficult for organic ions; no topography.
Infrared Photo-induced Force Microscopy (IR-PiFM, this work)	**Photothermal force**	**~10 nm**	**Yes (Height)**	**Yes (A-site cations, e.g., MA^+, FA^+)**	**Surface/Bulk (mode-selectabl e)**	Requires resonant IR absorption; limited to vibrational fingerprint regions.

migration in perovskites.

This table visually highlights that only IR-PiFM combines nanoscale spatial resolution (~10 nm), non-destructive chemical identification of specific A-site cations (MA^+/FA^+), and the tunable ability to probe both surface and bulk phenomena (via heterodyne/homodyne modes).

S3. SEM images of different mixed A-cation perovskite films

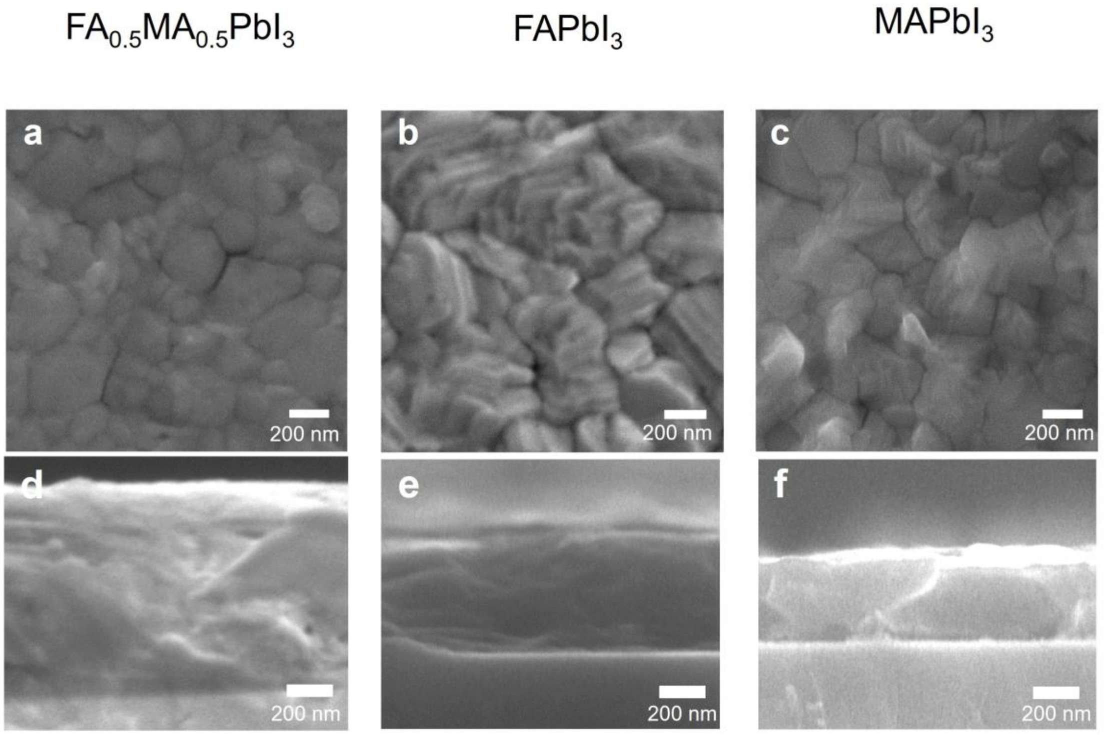

Figure S1. SEM images of different mixed-A cation perovskite films. (a-c) Top view SEM images of (a) $MAPbI_3$ film; (b) $FAPbI_3$ film; (c) $FA_{0.5}MA_{0.5}PbI_3$ film. (d-f) Cross sectional SEM images of (d) $MAPbI_3$ film; (e) $FAPbI_3$ film; (f) $FA_{0.5}MA_{0.5}PbI_3$ film.

The thickness of $MAPbI_3$, $FAPbI_3$ and $FA_{0.5}MA_{0.5}PbI_3$ perovskite films were around 400 ~700 nm according to the multiple SEM images.

S4. Steady-state photoluminescence (PL) spectra of different mixed A-cation perovskite films

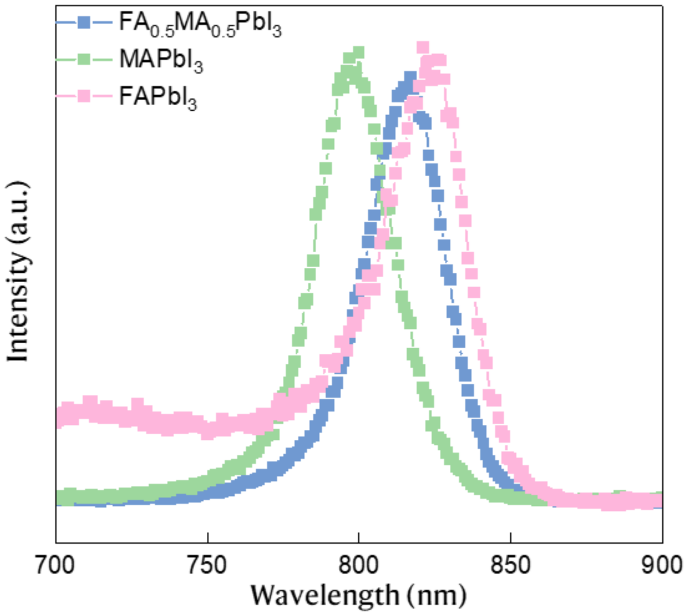

Figure S2. Steady-state photoluminescence (PL) spectra of $MAPbI_3$, $FAPbI_3$, and $FA_{0.5}MA_{0.5}PbI_3$, showing the bandgap difference between different mixed A-cation perovskite is not significant.

S5. Schematic illustration of the lateral device for PiFM measurement

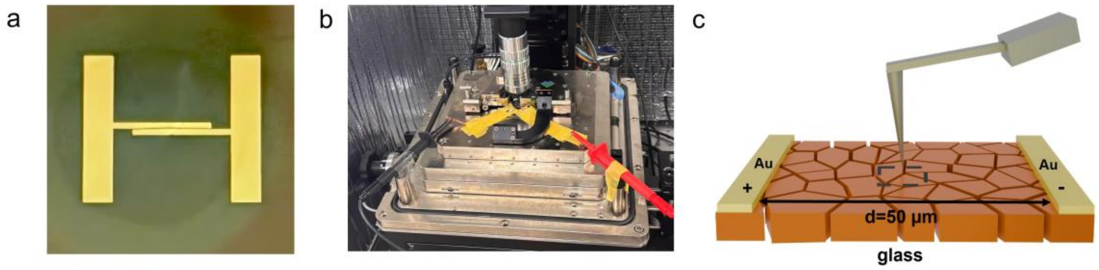

Figure S3. a) Photo image of a planar device with interdigitated gold electrodes spaced 50 μm apart. b) Photo image of the experimental setup (the AFM/PiFM system integrated with the electrical probing station). c) Schematic illustration of the lateral device for PiFM measurement.

S6. I-V curves of the different mixed A-cation perovskite devices obtained in dark and under illumination

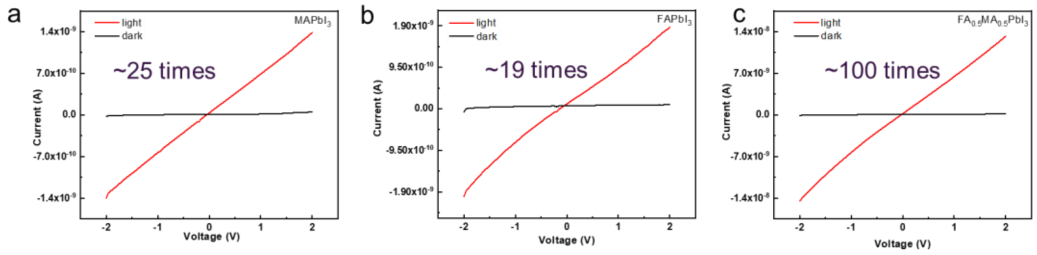

Figure S4. I-V curves of the (a)$MAPbI_3$, (b) $FAPbI_3$ and (c) $FA_{0.5}MA_{0.5}PbI_3$ perovskite lateral devices obtained in dark and under illumination.

S7. Ionic migration activation energy of different mixed A-cation perovskite

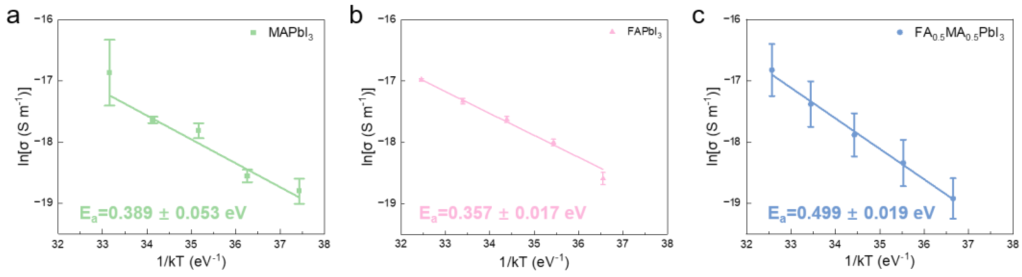

Figure S5. Ionic migration activation energy of (a) $MAPbI_3$; (b) $FAPbI_3$; (c) $FA_{0.5}MA_{0.5}PbI_3$. The curves are linearly fitted and details of the fitting are provided in experimental section. The error bars represent the standard deviations of the average ln(σ) values with 5 tests for each condition.

S8. Comparison of IR-PiFM spectra between grain boundaries and grains.

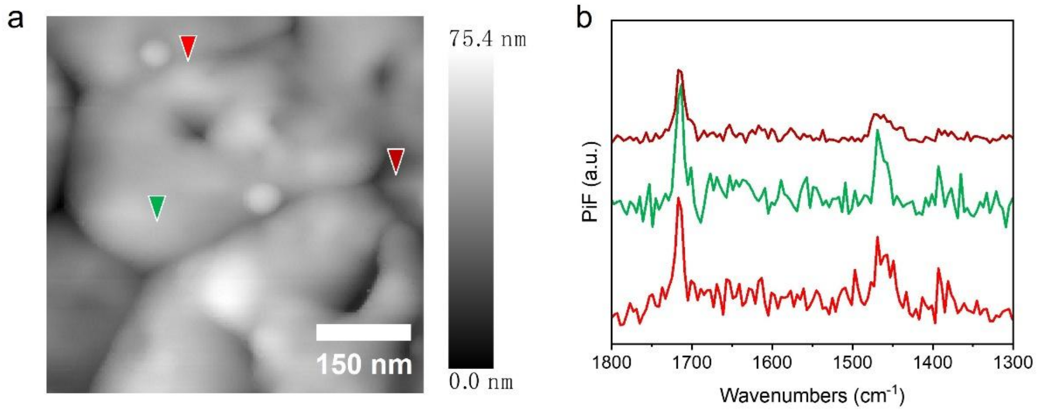

Figure S6. (a) Topography of a $FA_{0.5}MA_{0.5}PbI_3$. (b) IR-PiFM spectra measured at the marked point in (a).

S9. Signal acquisition frequency of temporal resolved IR-PiFM

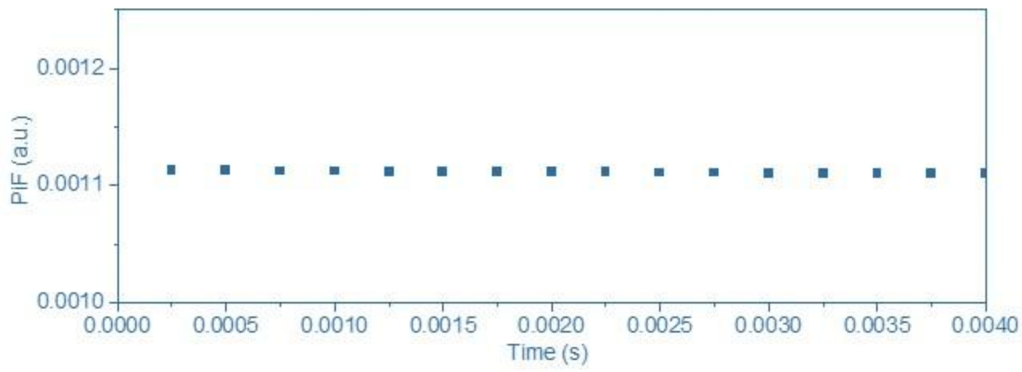

Figure S7. Randomly selected temporal resolved IR-PiFM signal. The signal acquisition frequency was 4000 Hz with temporal interval of 250 μs.

S10. Long term stability of $FA_{0.5}MA_{0.5}PbI_3$ perovskite device under IR-PiFM imaging

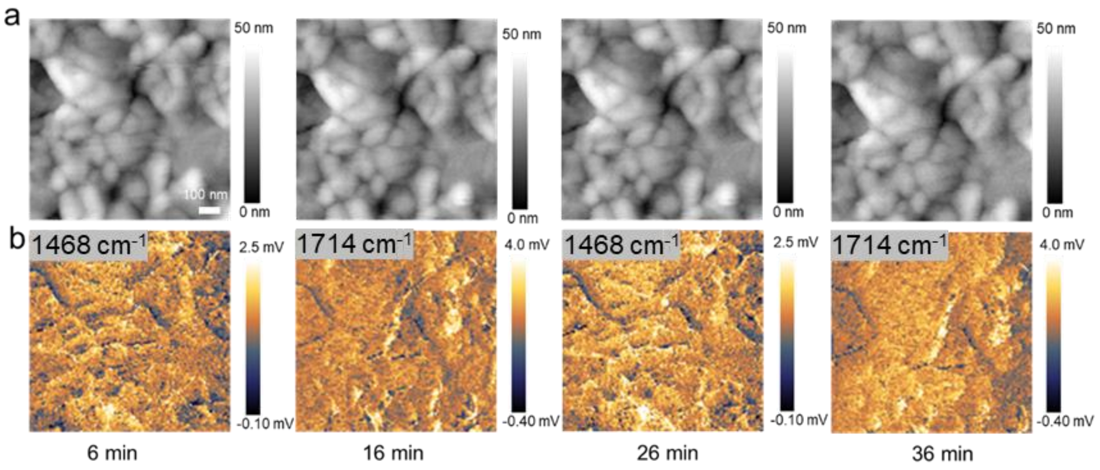

Figure S8. Time dependent evolution of (a) topography and (b) PiFM of a selected 1 μm × 1 μm area of $FA_{0.5}MA_{0.5}PbI_3$ device. Scale bar: 100 nm.

Since the mixed-cation perovskite devices are prone to phase separation under light exposure,[9] we conducted a series of experiments to confirm that the observed migration of FA^+ and MA^+ was caused by an external electric field. To eliminate other factors, we continuously imaged the mixed A-cation films without applying any bias. Under conditions consistent with the biased-experiments (using only natural light and an infrared laser), the film morphology and IR-PiF signal did not exhibit any changes during the 40-min continuous scanning process. These results suggest that the tip tapping and external light source stimulation did not cause deformation or cation migration in the mixed A-cation perovskite devices. Additionally, to rule out any potential influence on the infrared signal from switching the characteristic wavelength during imaging, we varied the scanning wavelength multiple times throughout the process. We found that the distribution and intensity of the cation signals remained unchanged, confirming the stability of our method.

S11. Long term stability of $FAPbI_3$ perovskite device under IR-PiFM imaging

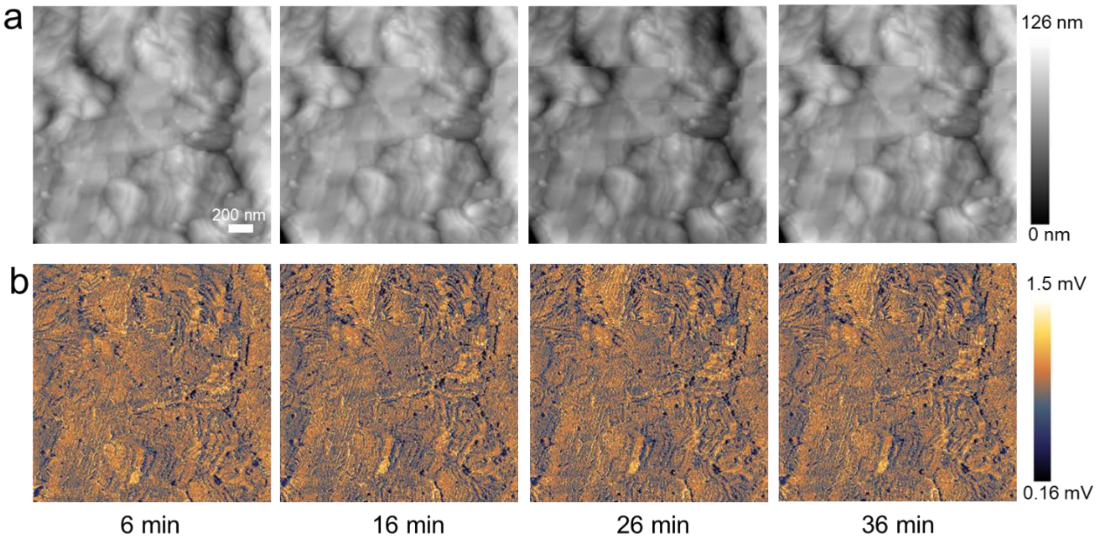

Figure S9. Time dependent evolution of (a) topography and (b) PiFM of a selected 2 µm × 2 µm area of $FAPbI_3$ device. Scale bar: 200 nm.

Within the 40 min imaging process, the morphology and IR-PiFM signals show negligible variation, indicating that the effects of tip tapping and IR laser illumination on the deformation and cation migration of $FAPbI_3$ perovskite device can be ignored.

S12. IR-PiF measurement in different laser power.

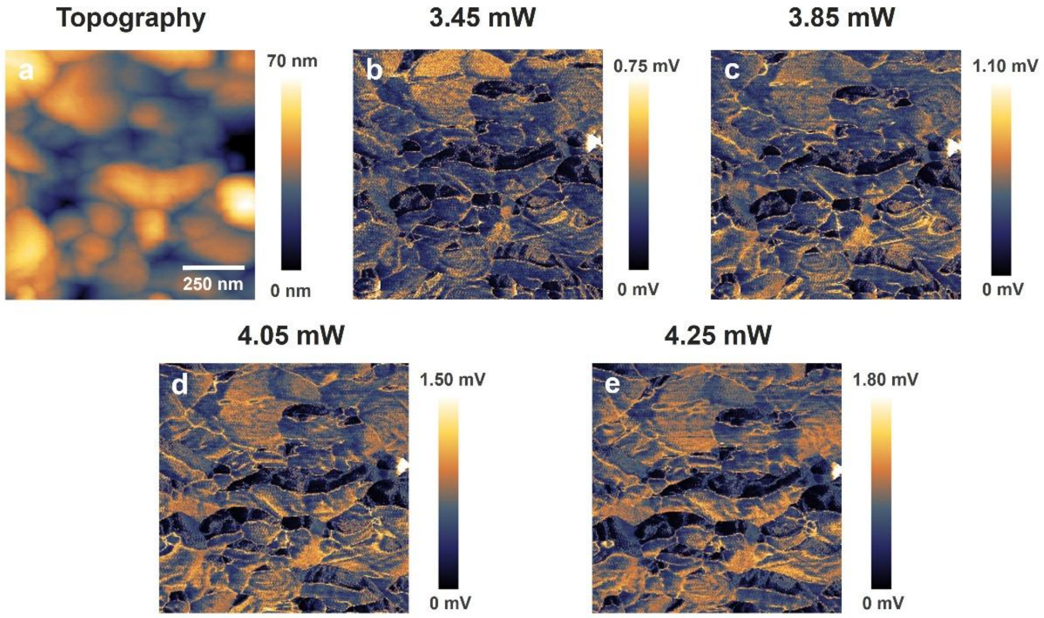

Figure S10. (a) Topography of $FAPbI_3$ device in a selected 1 μm × 1 μm area. (b-e) Laser power-dependent PiFM images.

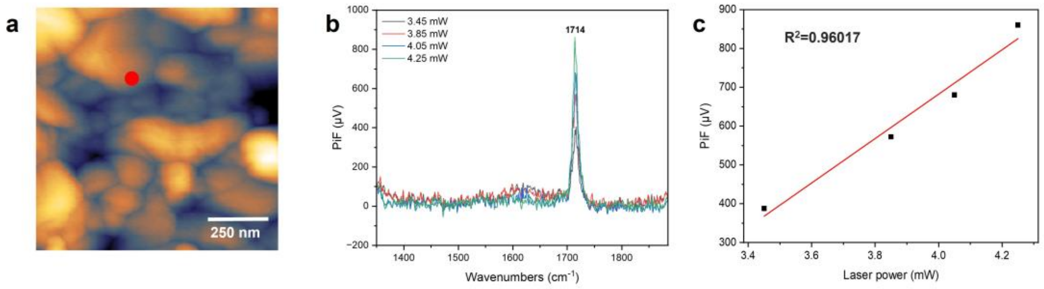

Figure S11. Laser power-dependent IR-PiFM characterization. (a) Topography of $FAPbI_3$ device in selected 1μm × 1 μm area; (b) PiF spectra at laser power ranging from 3.45 to 4.25 mW; (c) PiF signal intensity versus laser power at 1714 cm^{-1}.

The key results are summarized as follows:

Linear Power Dependence: The absolute PiFM signal intensity at the characteristic FA^+ peak (1714 cm^{-1}) scales linearly with laser power (Fig. S11c). This confirms the expected photothermal response without saturation or nonlinear effects that might indicate sample damage or anomalous heating.

Invariant Spatial Contrast: Crucially, as shown in the power-dependent PiFM images below (Fig. S10b-e), the relative spatial distribution of the IR signal (e.g., between grain interiors and boundaries) remains constant across all power levels.

These control experiments confirm that the cation migration patterns under electrical bias are genuine field-driven redistribution, not artifacts arising from the IR illumination itself. The linear response and stable contrast validate the reliability of our IR-PiFM measurements for quantitative comparison.

S13. Calculated diffusion coefficient and comparison

Table S2 Cation coefficient of different A-cation perovskite

Perovskite	Migration species	Method	Diffusion coefficient/$cm^2 \cdot s^{-1}$	Ref
**$MAPbI_3$**	**MA^+**	**PiFM**	**2.05×10^{-7}**	**This work**
$MAPbI_3$	MA^+	TID	1.67×10^{-10}	[3]
$MAPbI_3$	MA^+	DLTS	3.4×10^{-12}	[11]
$MAPbI_3$	MA^+	EIS	3.6×10^{-12}	[12]
**$FAPbI_3$**	**FA^+**	**PiFM**	**3.78×10^{-8}**	**This work**
$FAPbI_3$	FA^+	EIS	3.0×10^{-13}	[11]
**Mixed**	**MA^+**	**PiFM**	**1.36×10^{-8}**	**This work**
	**FA^+**		**3.25×10^{-9}**	
Mixed	MA^+/FA^+	EIS	2.0×10^{-12}	[12]

DLTS: Deep-level transient spectroscopy; EIS: Electrochemical impedance spectroscopy; TID: Transient ion drift.

The ion drifting velocity (v) of the cation at GB can be evaluated as,[13]

$$v = d/t \quad \text{(S-2)}$$

Thus, the ion mobility (μ) can be calculated as,

$$\mu = v/E \quad \text{(S-3)}$$

where the ion mobility (μ) is defined as the ratio between the ion drifting velocity (v) and electrical field (E). d is separation distance of two Au electrodes, t is the time when ion migration reaches equilibrium.

Then, the diffusion coefficient of cations at GBs can be calculated according to the Einstein Equations

$$D = k_B T \mu / q \quad \text{(S-4)}$$

D **is diffusion coefficient.** ***T*** **is the temperature,** k_B **is Boltzmann's constant,** ***q*** **is the amount of elementary charge carried.**

S14. Time-dependent IR-PiF signal curve on grain boundaries of different perovskite devices

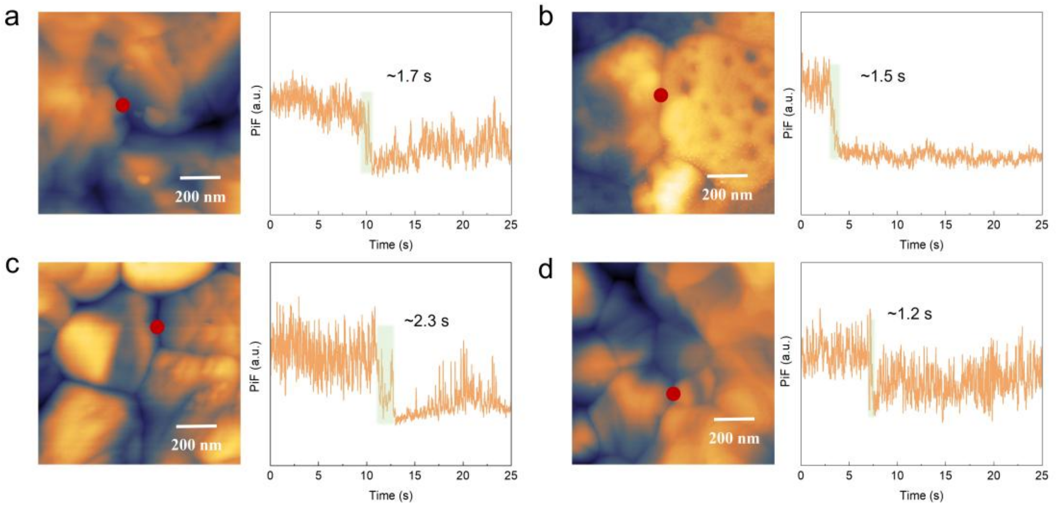

Figure S12. Topography and time-dependent IR-PiF signal curve at 1468 cm^{-1} of grain boundaries of 4 different $MAPbI_3$ devices. The signal curve was acquired at the position marked in topography images.

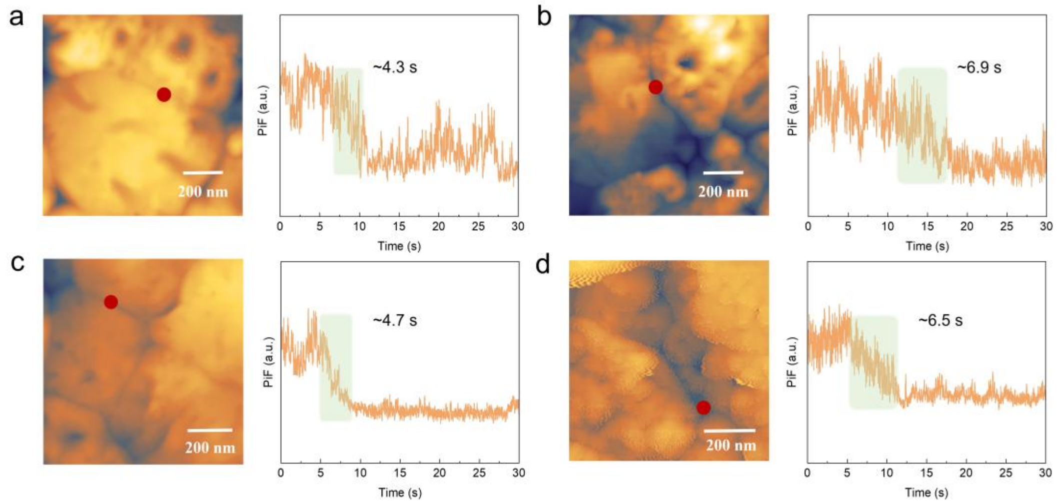

Figure S13. Topography and time-dependent IR-PiF signal curve at 1468 cm^{-1} of grain boundaries of 4 different $FA_{0.5}MA_{0.5}PbI_3$ devices. The signal curve was acquired at the position marked in topography images.

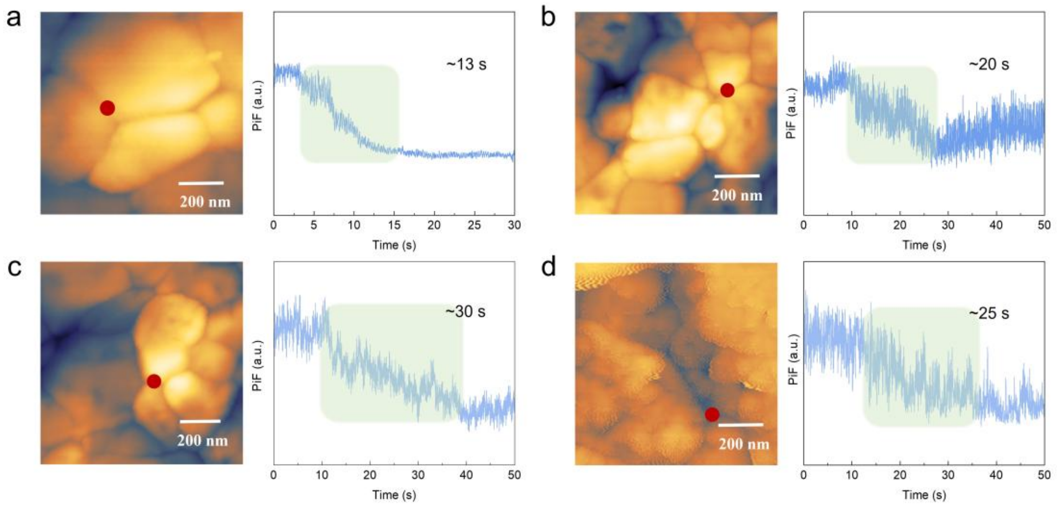

Figure S14. Topography and time-dependent IR-PiF signal curve at 1714 cm^{-1} of grain boundaries of 4 different $FA_{0.5}MA_{0.5}PbI_3$ devices. The signal curve was acquired at the position marked in topography images.

S15. Calculation and statistics of diffusion coefficient

Table S3 Cation diffusion coefficient of different A-cation perovskites

Perovskite	Migration species	Migration time/s	Diffusion coefficient/$cm^2 \cdot s^{-1}$	Average/$cm^2 \cdot s^{-1}$
		1.5	2.14×10^{-7}	
		1.7	1.89×10^{-7}	
$MAPbI_3$	MA^+	1.5	2.14×10^{-7}	$(2.05 \pm 0.47) \times 10^{-7}$
		2.3	1.39×10^{-7}	
		1.2	2.68×10^{-7}	
		3.2	2.01×10^{-8}	
		4.3	1.49×10^{-8}	
$FA_{0.5}MA_{0.5}PbI_3$	MA^+	6.9	0.93×10^{-8}	$(1.36 \pm 0.44) \times 10^{-8}$
		4.7	1.36×10^{-8}	
		6.5	0.99×10^{-8}	
		19	3.39×10^{-9}	
		13	4.94×10^{-9}	
$FA_{0.5}MA_{0.5}PbI_3$	FA^+	20	3.21×10^{-9}	$(3.25 \pm 1.07) \times 10^{-9}$
		30	2.14×10^{-9}	
		25	2.57×10^{-9}	

The corresponding diffusion coefficient was calculated based on the results in S11. The results obtained on the same type perovskite exhibit high consistency, indicating that the robustness and generality of IR-PiF method.

S16. Time-dependent IR-PiF signal curve on grains of different perovskite devices

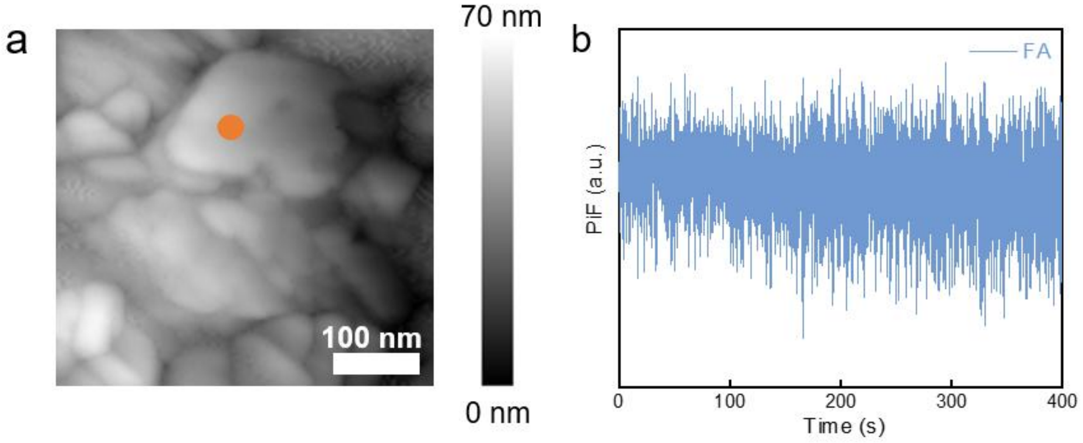

Figure S15. (a) AFM image of $FAPbI_3$ perovskite. Scale bar: 100 nm. (b) Time-dependent IR-PiF signal curve of the position on grain marked in (a) based on $FAPbI_3$ perovskite under 2 V bias.

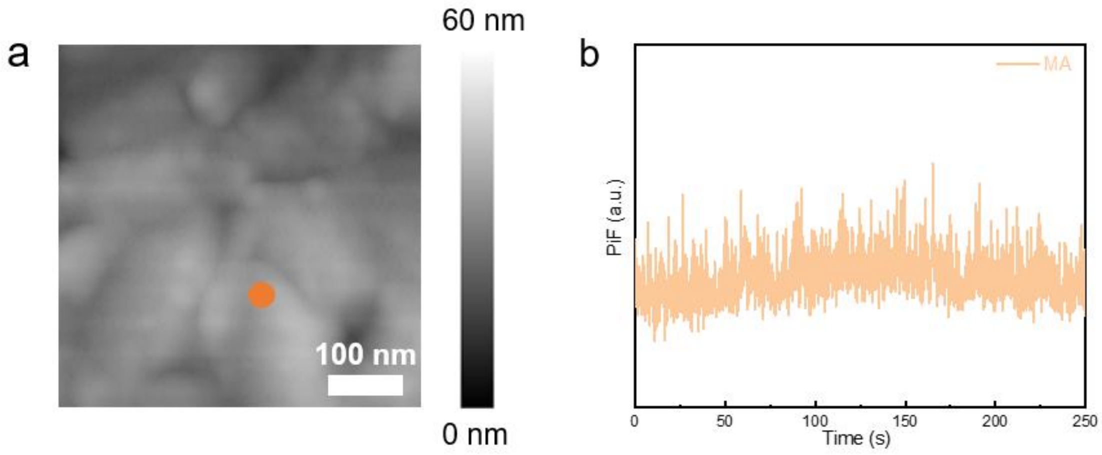

Figure S16. (a) AFM image of $MAPbI_3$ perovskite. Scale bar: 100 nm. (b) Time-dependent IR-PiF signal curves of the position on grain marked in (a) based on $MAPbI_3$ perovskite under 2 V bias.

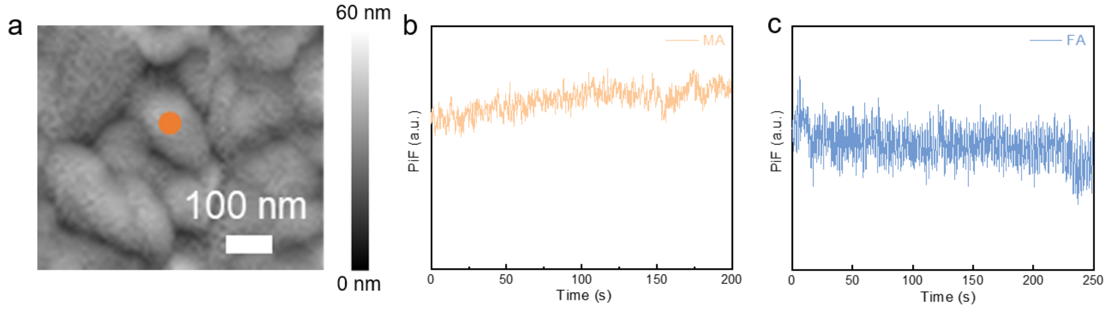

Figure S17. (a) AFM image of $FA_{0.5}MA_{0.5}PbI_3$ perovskite. Scale bar: 100 nm. (b, c) Time-dependent IR-PiF signal curves of the position on grain marked in (a) based on $FA_{0.5}MA_{0.5}PbI_3$ perovskite under 10 V bias at (b)1468 cm^{-1} and (c) 1714 cm^{-1}.

S17. XRD patterns of different A-cation perovskite

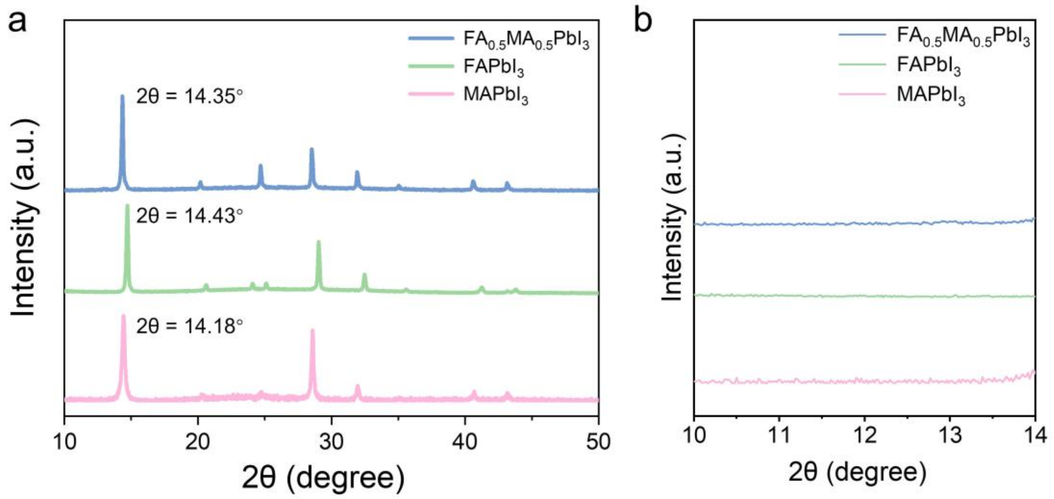

Figure S18. (a) XRD patterns of different A-site cations perovskite films. (b) More detailed XRD patterns from 10 to 14.

The $MAPbI_3$ film exhibited a typical tetragonal phase with most grains oriented near the <110>$_t$ direction. The $FAPbI_3$ and $FA_{0.5}MA_{0.5}PbI_3$ films showed characteristic peaks corresponding to the cubic α-phase at (100), (110), and (111) planes. The prominent (111) peaks with varying intensities suggest the formation of $\{111\}_c$ twin boundaries with different orientations. Furthermore, no peaks at 11.8 °—characteristic of the δ-phase (hexagonal) $FAPbI_3$—were observed, confirming the α-phase of the $FAPbI_3$ and $FA_{0.5}MA_{0.5}PbI_3$ films. Considering the ionic radii of FA^+ (253 pm) and MA^+ (217 pm), the composition-engineering reduced the average ionic radius of the A-site cations, leading to smaller lattice parameters.

S18. DFT simulations for cation migration

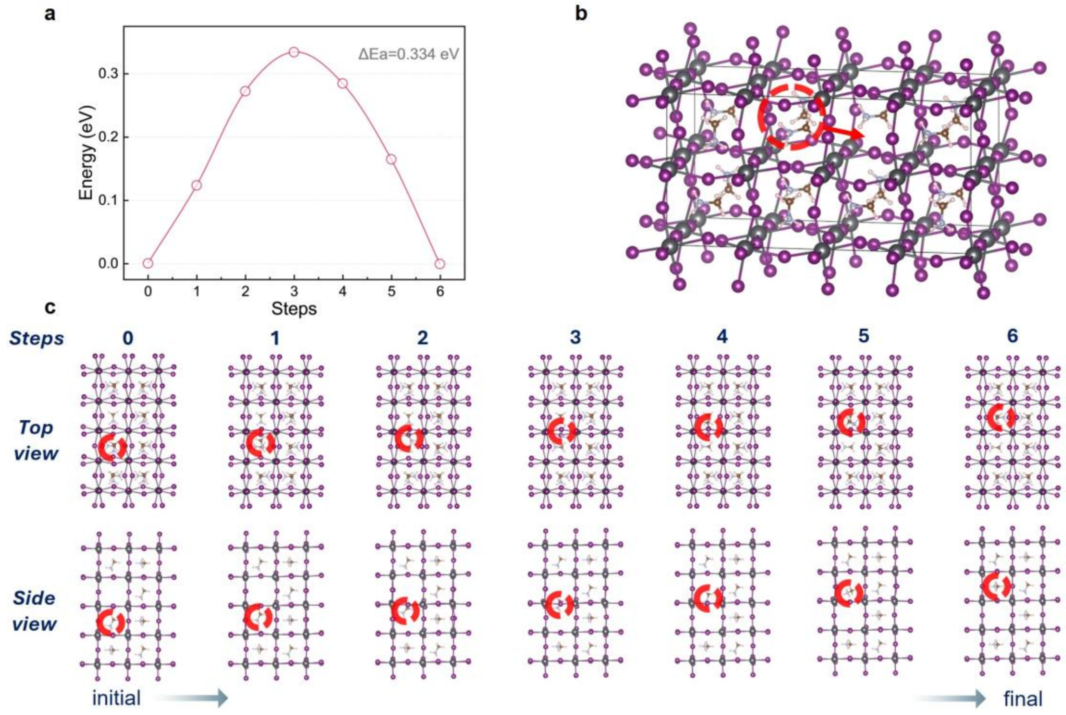

Figure S19. (a) Energy profiles of MA^+ migration to adjacent lattice sites in $MAPbI_3$ along the migration paths. (b) 3D schematic diagram of ion migration of MA^+. (c) Structural images of the optimized migration paths for MA^+.

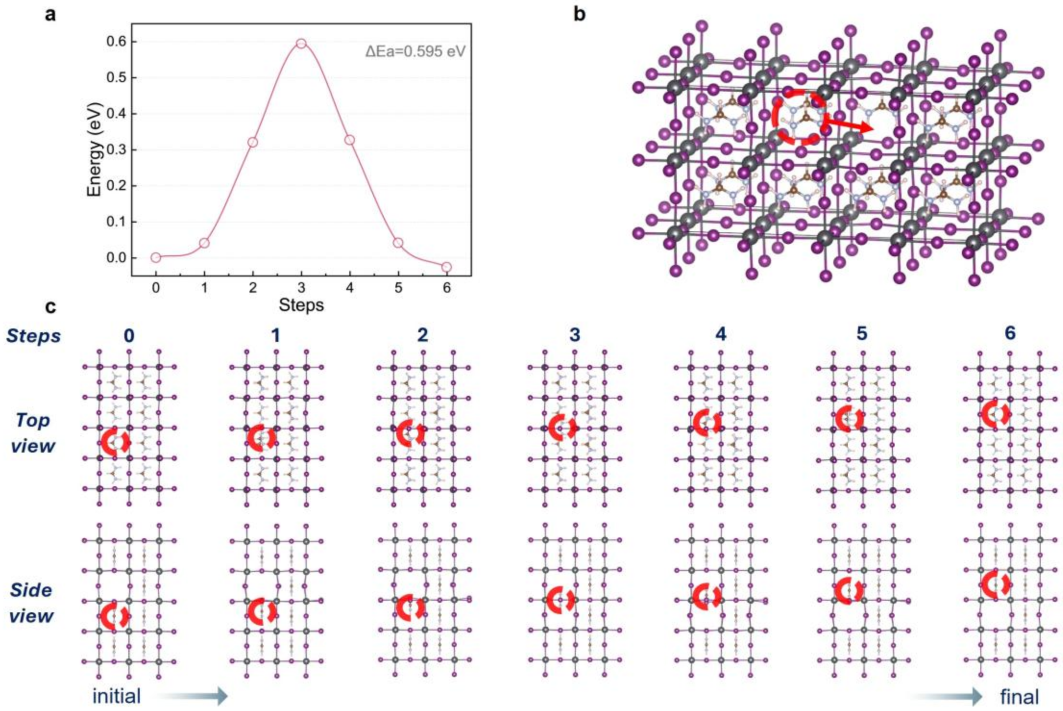

Figure S20. (a) Energy profiles of FA^+ migration to adjacent lattice sites in $FAPbI_3$ along the migration paths. (b) 3D schematic diagram of ion migration of FA+. (c) Structural images of the optimized migration paths for FA^+.

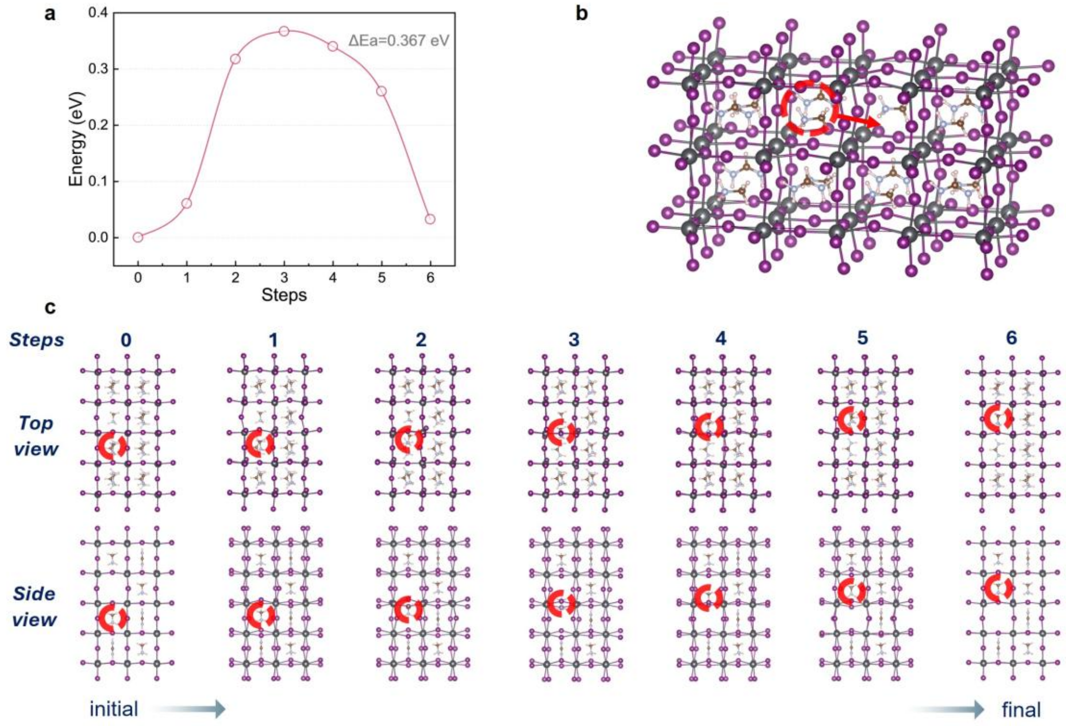

Figure S21. (a) Energy profiles of MA^+ migration to adjacent lattice sites in $FA_{0.5}MA_{0.5}PbI_3$ along the migration paths. (b) 3D schematic diagram of ion migration of MA^+. (c) Structural images of the optimized migration paths for MA^+.

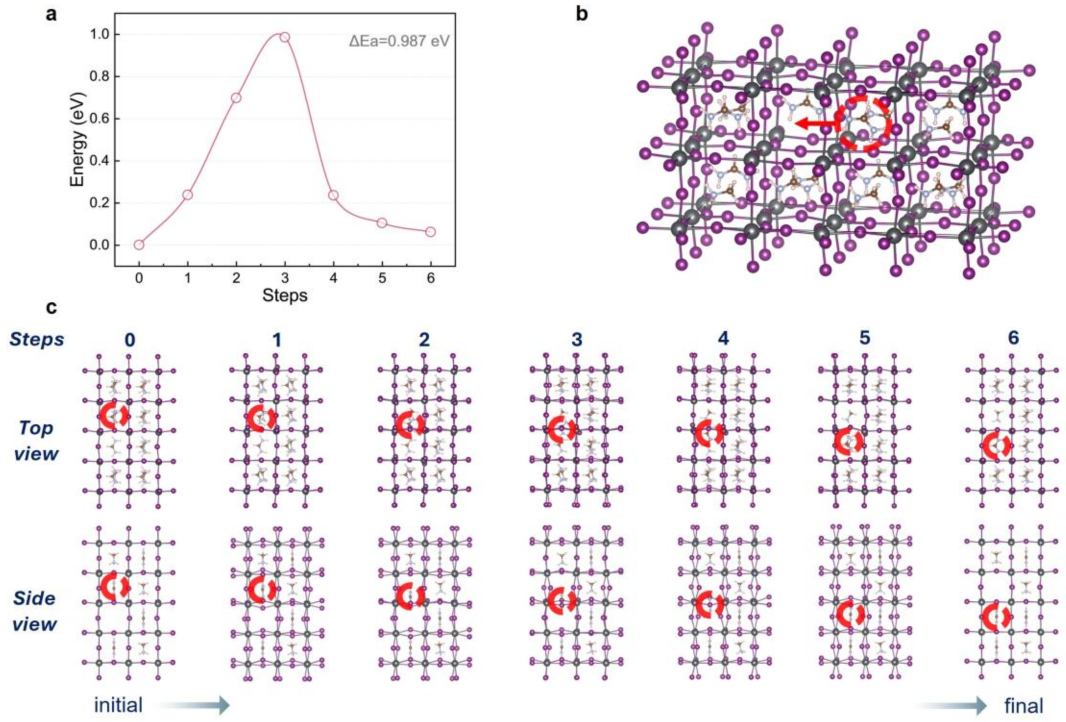

Figure S22. (a) Energy profiles of FA^+ migration to adjacent lattice sites in $FA_{0.5}MA_{0.5}PbI_3$ along the migration paths. (b) 3D schematic diagram of ion migration of FA^+. (c) Structural images of the optimized migration paths for FA^+.

S19. Time dependent IR-PiFM images of $FAPbI_3$ device under bias

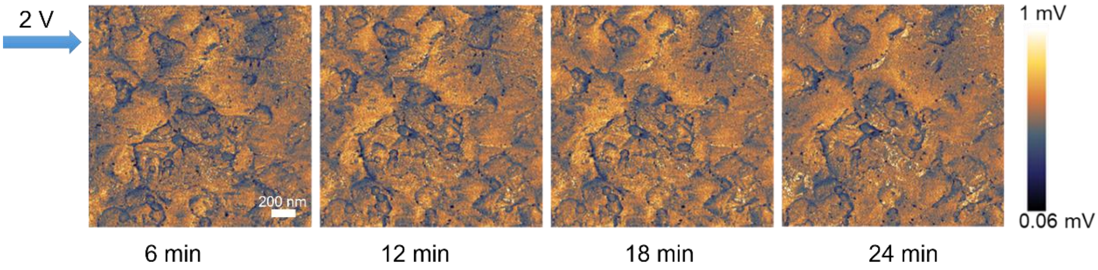

Figure S23. IR-PiFM images of $FAPbI_3$ device under 2 V bias with time.

Time-dependent IR-PiFM images of the $FAPbI_3$ device under a 2 V bias reveal that the migration nearly reaches equilibrium on grain boundaries (GBs) after applying a 3-minute bias. Subsequent images do not show discernible difference. This observation may be attributed to the significantly faster migration of cations in GBs, which was reported to be 2 to 4 orders of magnitude faster than grain migration.[10]

S20. Bias cycling experiment of $FAPbI_3$ device

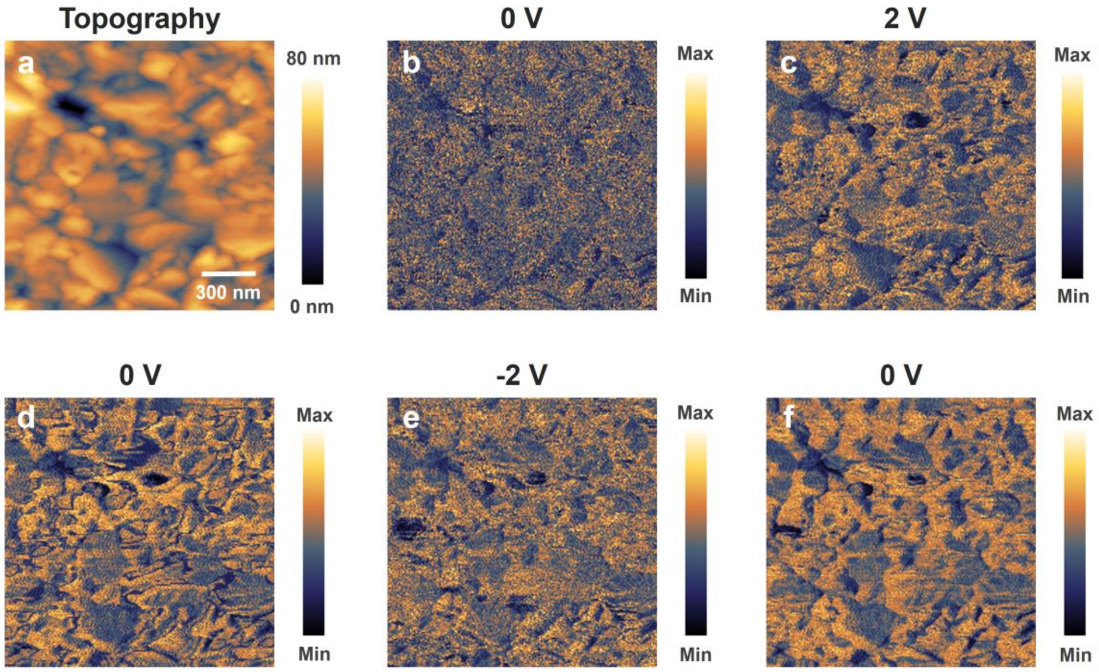

Figure S24. Bias cycling experiment and corresponding IR-PiFM images of $FAPbI_3$ device. (a) Topography of $FAPbI_3$ device in selected 1.5 μm × 1.5 μm area; (b-f) PiF images in the range of -2 V to 2 V.

To directly assess the reversibility of cation migration, we have performed multi-round bias cycling experiments.Briefly, we applied a sequence of biases (e.g., 0 V → +2 V → 0 V → -2 V → 0 V) to an $FAPbI_3$ device while monitoring the FA^+ distribution via IR-PiFM.

We found that FA^+ migration exhibits significant kinetic hysteresis. Once redistributed by an applied bias, the FA^+ ions do not fully revert to their original distribution upon bias removal within our experimental timeframe. This irreversibility has an important methodological implication: it precludes the reliable study of multiple, sequential bias cycles on the exact same grain boundary location, as the initial state cannot be restored.

This inherent irreversibility is precisely why our core experimental strategy—measuring the first-response kinetics and steady-state redistribution at multiple, pristine grain boundaries across different devices (as shown in Figs. 2, 4, and SI Sections S12, S13)—is both necessary and robust. It ensures that each

measurement captures the intrinsic, initial response of a fresh site to the applied field, avoiding confounding effects from prior ion-drift history. The excellent consistency we observed across multiple independent sites (see statistical data in Table S2) confirms the reliability of this approach.

This result provides an important complementary perspective. It reinforces the distinct kinetic behavior between FA^+ and MA^+. FA^+ migration is more “locked-in” after drift, consistent with its higher calculated activation energy and stronger interaction with the lattice. This further underscore the cation-specific dynamics that our IR-PiFM approach is able to resolve.

S21. IR-PiFM images of different perovskite devices before and after applying bias

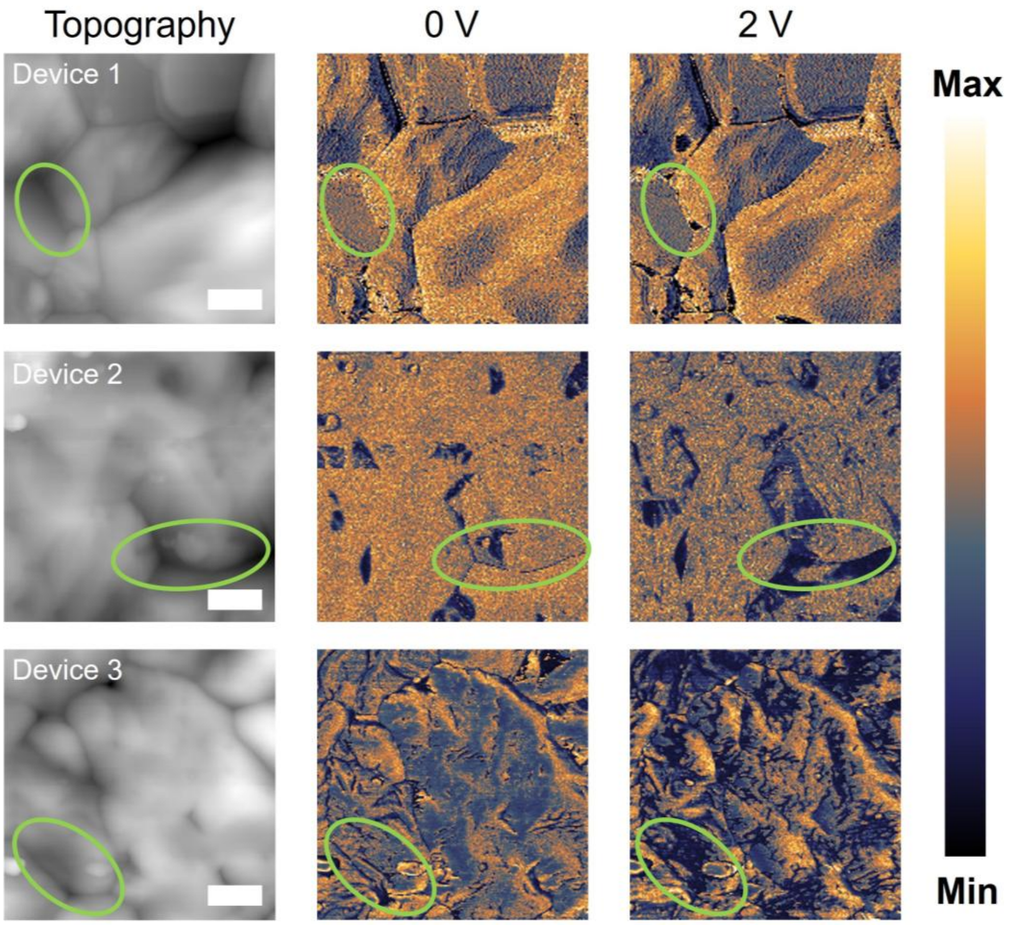

Figure S25. IR-PiFM images of three different $MAPbI_3$ devices before and after applying bias. Green elliptical circles mark the grain boundaries and the PiF signal change in different $MAPbI_3$. All images were acquired at 1468 cm^{-1}. Scale bar: 200 nm.

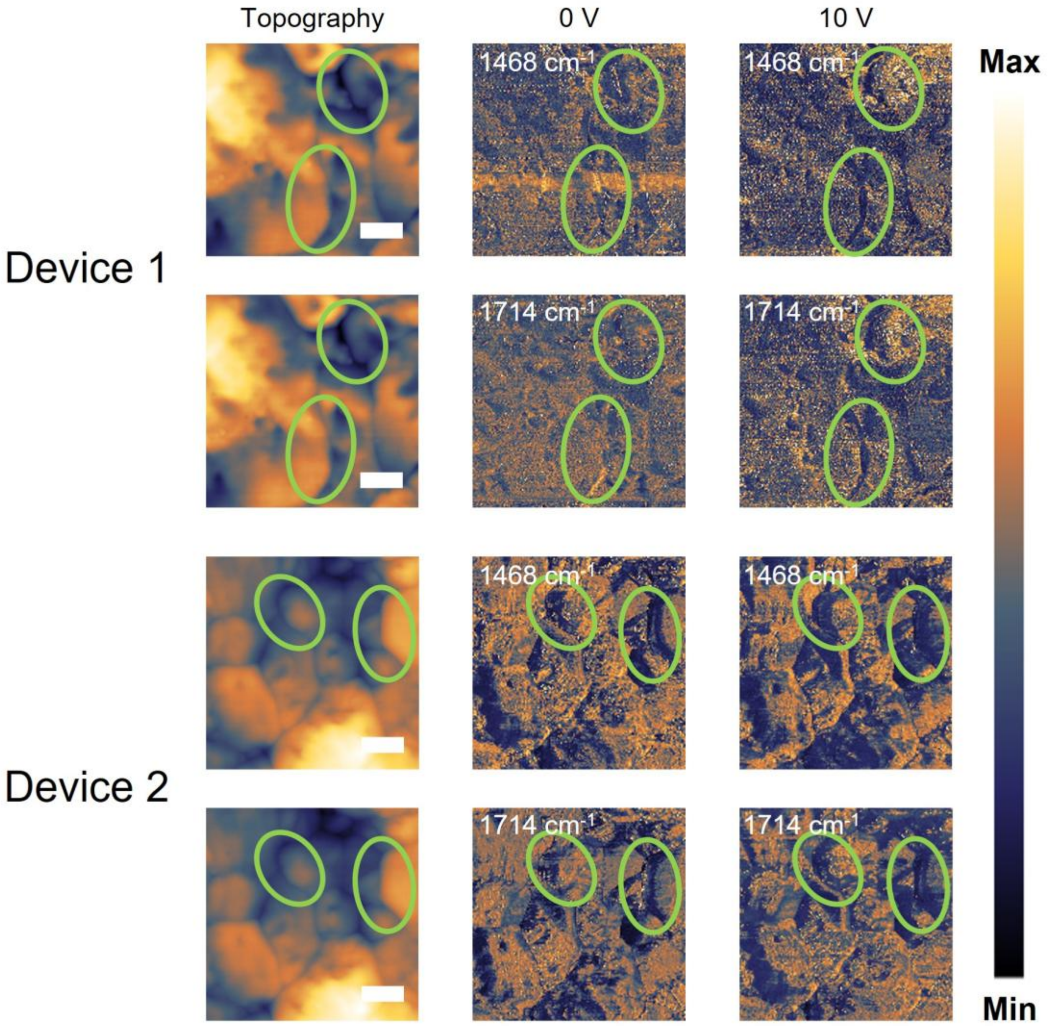

Figure S26. IR-PiFM images of two different $FA_{0.5}MA_{0.5}PbI_3$ devices before and after applying bias. Both of the two devices were imaged at 1468 and 1714 cm^{-1}. Green elliptical circles mark the grain boundaries and the PiF signal change in different $FA_{0.5}MA_{0.5}PbI_3$. Scale bar: 200 nm.

S22. Line profile of AFM and external electric field dependent PiFM signals of cations in $FA_{0.5}MA_{0.5}PbI_3$ perovskite device

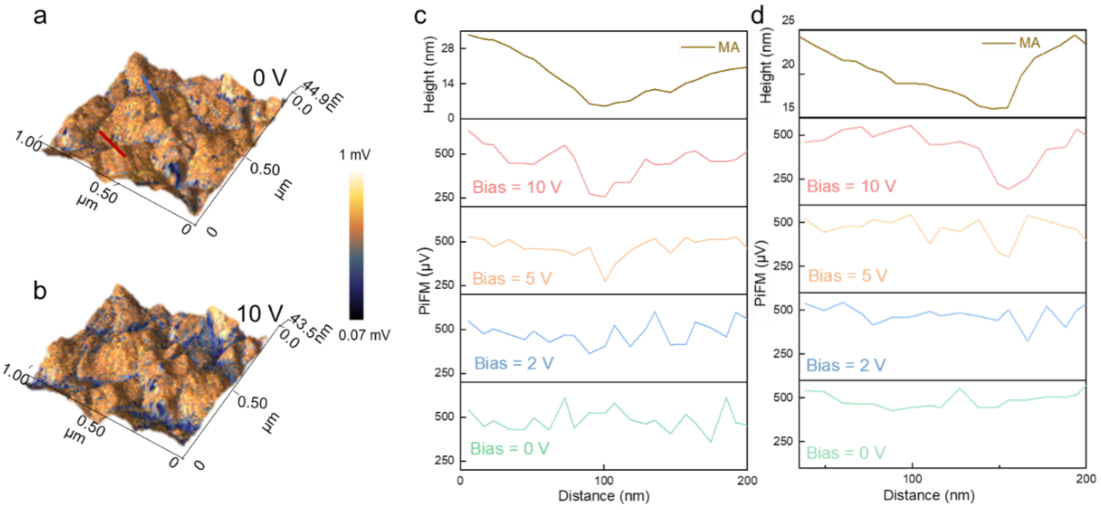

Figure S27. Imaging of electric field induced MA^+ migration via homodyne mode. (a, b) Merged AFM and homodyne mode IR-PiFM images at 1468 cm^{-1} of MA^+ obtained under a bias of 0 V (a) and 10V (b). (c-d) Two line-profiles of the AFM and external electric field dependent PiFM signals along the red lines (c) and blue lines (d) indicated in (a).

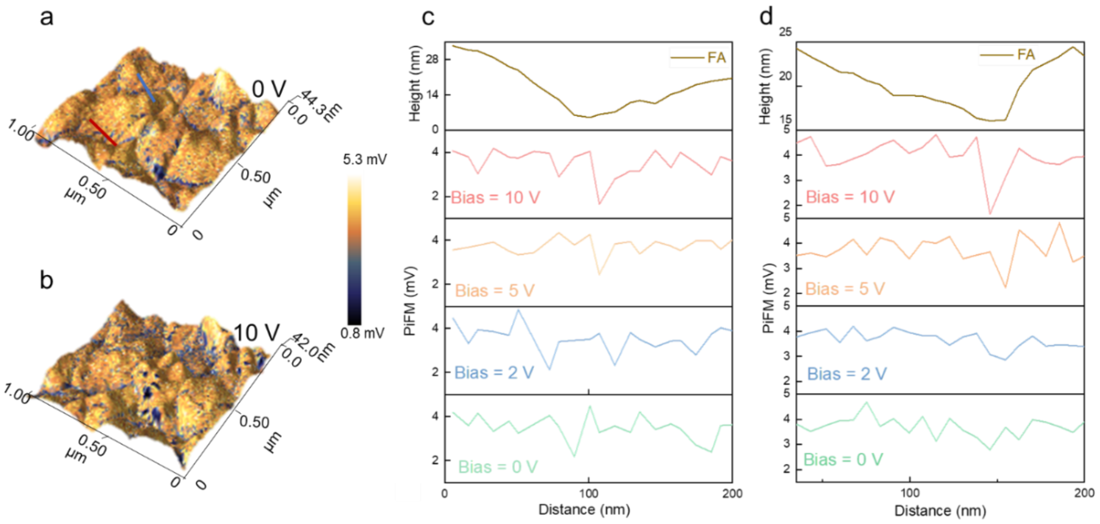

Figure S28. Imaging of electric field induced FA^+ migration via homodyne mode. **(**a, b) Merged AFM and homodyne mode IR-PiFM images at 1714 cm^{-1} of FA^+ obtained under a bias of 0 V (a) and 10V (b). (c-d) Two line-profiles of the AFM and external electric field dependent PiFM signals along the red lines (c) and blue lines (d) indicated in (a).

S23. Imaging of electric field induced cation migration on $FA_{0.5}MA_{0.5}PbI_3$ perovskite device via heterodyne mode

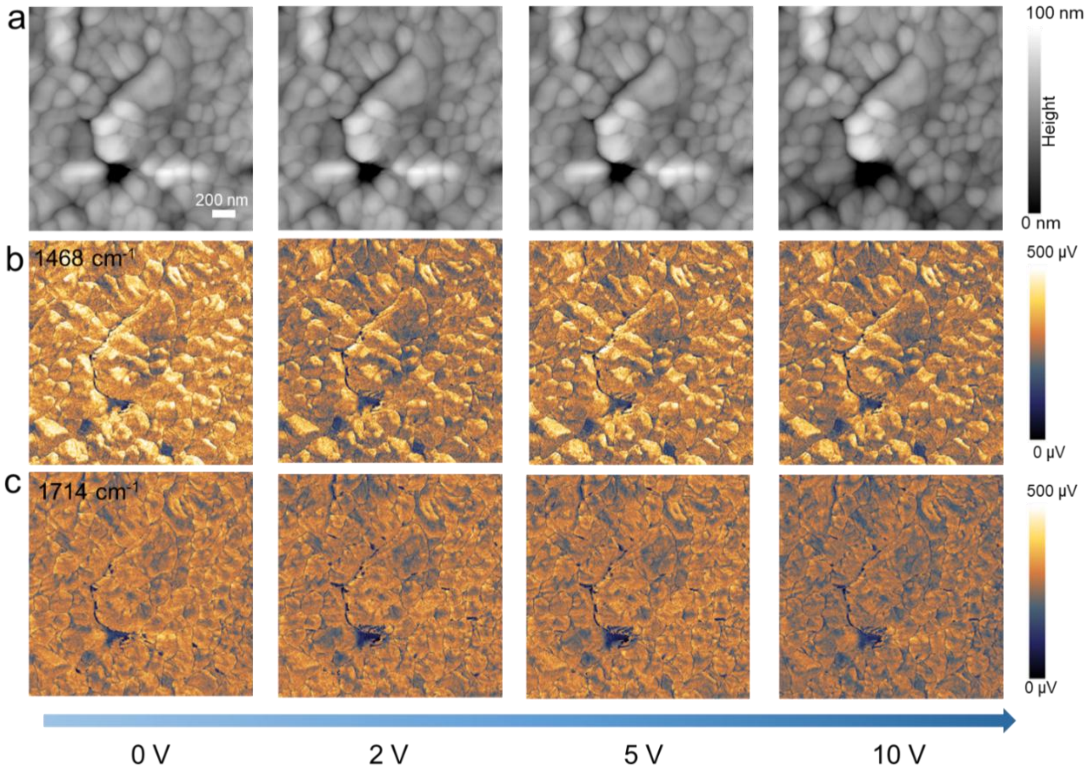

Figure S29. Imaging of electric field induced cation migration on $FA_{0.5}MA_{0.5}PbI_3$ perovskite device via heterodyne mode. (a) AFM images under bias from 0 V, 2 V, 5 V and 10 V. (b, c) Bias-dependent heterodyne mode IR-PiFM images at (b) 1468 cm^{-1} and (c) 1714 cm^{-1}. Scale bar: 200 nm.

S24. Scheme of the set up for V_{OC} measurements

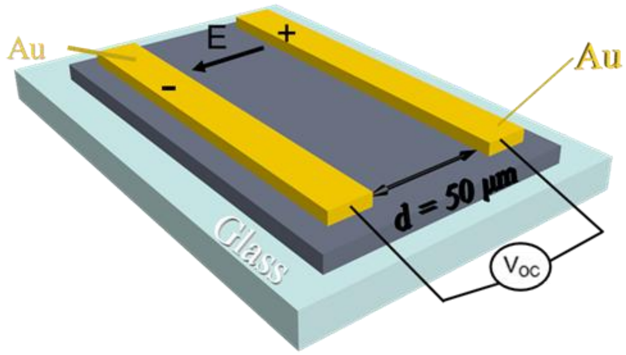

Figure S30. Schematic diagram of V_{OC} measurement under polarized electric field.

To measure the V_{OC}, the perovskite devices were placed horizontally under different electric fields and polarized for 100 s. Then, the bias was removed and the potential difference (V_{OC}) between two Au electrodes was measured.

References

[1] Li, W.; Rothmann, M. U.; Zhu, Y.; Chen, W.; Yang, C.; Yuan, Y.; Choo, Y. Y.; Wen, X.; Cheng, Y.-B.; Bach, U.; et al. The critical role of composition-dependent intragrain planar defects in the performance of $MA_{1-x}FA_xPbI_3$ perovskite solar cells. *Nature Energy* **2021**, 6 (6), 624-632.

[2] Bai, Y.; Huang, Z. J.; Zhang, X.; Lu, J. Z.; Niu, X. X.; He, Z. W.; Zhu, C.; Xiao, M. Q.; Song, Q. Z.; Wei, X. Y.; et al. Initializing film homogeneity to retard phase segregation for stable perovskite solar cells. *Science* **2022**, 378 (6621), 747-754.

[3] Li, B.; Kan, C.; Hang, P.; Fang, Y.; Zuo, L.; Song, L.; Zhang, Y.; Yang, D. and Yu, X. Understanding the Influence of Cation and Anion Migration on Mixed-Composition Perovskite Solar Cells via Transient Ion Drift. *Physica Status Solidi-Rapid Research Letters* **2021**, 15, 2100225.

[4] Sifat, A. A.; Jahng, J.; Potma, E. O. Photo-induced force microscopy (PiFM) – principles and implementations. *Chemical Society Reviews* **2022**, 51, 4208-4222.

[5] Shcherbakov, M. R.; Potma, E. O.; Sugawara, Y.; Nowak, D.; Stepanova, M.; Davies, P. R.; Davies-Jones, J.; Wickramasinghe, H. K. *Nature Reviews Methods Primers* **2025**, 5, 34.

[6]Kresse, G.; Joubert, D. From ultrasoft pseudopotentials to the projector augmented-wave method. *Physical Review B* **1999**, 59, 1758-177.

[7] Perdew, J. P.; Burke, K.; Ernzerhof, M. Generalized gradient approximation made simple. *Physical Review Letters* **1996**, 77, 3865-3868.

[8] Grimme S, Antony J, Ehrlich S and Krieg H. A consistent and accurate ab initio parametrization of density functional dispersion correction (DFT-D) for the 94 elements H-Pu *Journal of Chemical Physics* **2010**, 132, 154104.

[9] Noh, J. H.; Im, S. H.; Heo, J. H.; Mandal, T. N.; Seok, S. I. Chemical management for colorful, efficient, and stable inorganic–organic hybrid nanostructured solar cells. *Nano Letters* **2013**, 13 (4), 1764-1769.

[10] Ghasemi, M.; Guo, B.; Darabi, K.; Wang, T.; Wang, K.; Huang, C. W.; Lefler, B. M.; Taussig, L.; Chauhan, M.; Baucom, G.; et al. A multiscale ion diffusion framework sheds light on the diffusion-stability-hysteresis nexus in metal halide

perovskites. *Nature Materials* **2023**, 22 (3), 329-337.

[11] Futscher, M.; Lee, J.; McGovern, L.; Muscarella, L.; Wang, T.; Haider, M.; Fakharuddin, A.; Schmidt-Mende, L. and Ehrler, B. Quantification of ion migration in CH3NH3PbI3 perovskite solar cells by transient capacitance measurements. *Materials Horizons* **2019**, 6, 1497-1503.

[12] Bag, M.; Renna, A.; Adhikari, R.; Karak, S.; Liu, F.; Lahti, P.; Russell, T.; Tuominen, M. and Venkataraman, D. Kinetics of Ion Transport in Perovskite Active Layers and Its Implications for Active Layer Stability. *Journal of the American Chemical Society* **2015**, 137 (40), 13130–13137.

[13] Yuan, Y.; Chae, J.; Shao, Y.; Wang, Q.; Xiao, Z.; Centrone, A.; Huang, J. Photovoltaic switching mechanism in lateral structure hybrid perovskite solar cells. *Advanced Energy Materials* **2015**, *5* (15), 1500615.
